# Synthesis and Biological Evaluation of Apigenin Derivatives as Antibacterial and Antiproliferative Agents

**DOI:** 10.3390/molecules180911496

**Published:** 2013-09-17

**Authors:** Rui Liu, Hongchi Zhang, Maosen Yuan, Jiao Zhou, Qin Tu, Jian-Jun Liu, Jinyi Wang

**Affiliations:** 1 Colleges of Science and Forestry, Northwest A&F University, Yangling 712100, Shaanxi, China; 2 College of Agronomy and Life Sciences, Shanxi Datong University, Datong 037009, Shanxi, China

**Keywords:** synthesis, biological evaluation, apigenin derivatives, antibacterial activity, antiproliferative activity, MTT assay

## Abstract

Two series of apigenin [5,7-dihydroxy-2-(4-hydroxyphenyl)-4H-chromen-4-one] derivatives, **3a**–**3j** and **4a**–**4j**, were synthesized. The apigenin and alkyl amines moieties of these compounds were separated by C_2_ or C_3_ spacers, respectively. The chemical structures of the apigenin derivatives were confirmed using ^1^H-NMR, ^13^C-NMR, and electrospray ionization mass spectroscopy. The *in vitro* antibacterial and antiproliferative activities of all synthesized compounds were determined. Among the tested compounds, **4a**–**4j** displayed significant antibacterial activity against the tested strains (*Staphylococcus aureus*, *Bacillus subtilis*, *Escherichia coli*, and *Pseudomonas aeruginosa*). Additionally, **4i** showed the best inhibitory activity with minimum inhibitory concentrations of 1.95, 3.91, 3.91, and 3.91 μg/mL against *S. aureus*, *B. subtilis*, *E. coli*, and *P. aeruginosa*, respectively. The antiproliferative activity of the apigenin derivatives was evaluated by an MTT [3-(4,5-dimethylthiazol-2-yl)-2,5-diphenyl tetrazolium bromide] assay. We determined that **4a**–**4j** displayed better growth inhibition activity against four human cancer cell lines, namely, human lung (A549), human cervical (HeLa), human hepatocellular liver (HepG2), and human breast (MCF-7) cancer cells, than the parent apigenin. Compound **4j** was found to be the most active antiproliferative compound against the selected cancer cells. Structure-activity relationships were also discussed based on the obtained experimental data.

## 1. Introduction

The battle against cancer is one of the biggest social problems encountered worldwide, because cancer is one of the most dreadful diseases, and the second leading cause of death in developing as well as developed countries [1,2]. The World Health Organization reported that cancer accounted for 7.6 million deaths (around 13% of all deaths) in 2008, and 13.1 million deaths is estimated for 2030. In the past few decades, various drugs have been developed in cancer research to reduce mortality and improve survival of cancer patients. However, the majority of these studies failed because of low selectivity and frequency of adverse events. Therefore, the development of novel anticancer agents that selectively act on the target without side effects has become a primary objective for medicinal chemists [3]. A dramatic increase in the investigation of naturally occurring products in terms of their potential for treating cancers is recently observed [4]. One promising group of natural products for cancer therapy comprises the flavonoids. These compounds play an important role in cancer treatment. Multiple epidemiological studies have reported that flavonoids can reduce or prevent the incidence of various cancers [5,6]. For instance, quercetin was reported to induce apoptosis via the mitochondrial pathway in KB and KBv200 cells [7]. Valdameri *et al*. discovered that chalcones are selective inhibitors of the breast cancer resistance protein [8].

Flavonoids are important natural products that are universally found in fruits, cereals, legumes, vegetables, seeds, spices, and medicinal plants [9]. Apigenin (**1**), a type of flavonoid, is abundant in various fruits, vegetables, and medicinal plants, such as parsley, onion, orange, paper mulberry, *Veronica linariifolia*, and *Rhizoma Polygoni Cuspidati* [ 10]. Apigenin is widely distributed and also found to be useful as pharmaceutical agents. In addition to its anti-inflammatory and antioxidation activities, apigenin has been used as a dietary supplement because of its anticancer properties [11]. Previous studies have shown that apigenin has anticancer activity in numerous human cancer cells, such as prostate cancer, colon carcinoma, and breast cancer, with low cytotoxicity and no mutagenic activity [12].

Multi-drug resistance is drawing much attention in recent years because of the extensive use of antibiotics. The resistant strains curtail the life span of drugs. These shortcomings lead to an urgent global need for finding new antimicrobial drugs, particularly from natural resources [13]. Naturally derived apigenin was reported to have potential antimicrobial activity [14]. Meanwhile, various compounds possessing aminoalkylation at the 7-O position have been shown to have significant biological activities [15,16]. In view of these findings and in continuation of our study on apigenin, we report the synthesis of two series of apigenin derivatives, in which the apigenin ring system is linked to different amines separated by 2-carbon or 3-carbon spacers at C-7 position to enhance their lipophilicity. The antiproliferative activities of these derivatives were tested against four human cancer cell lines, namely, the human lung (A549), human cervical (HeLa), human hepatocellular liver (HepG2), and human breast (MCF-7) cancer cells, using a standard 3-(4,5-dimethylthiazol-2-yl)-2,5-diphenyl tetrazolium bromide (MTT) assay. The antibacterial activities of these derivatives were also investigated.

## 2. Results and Discussion

### 2.1. Chemistry

**Scheme 1:**
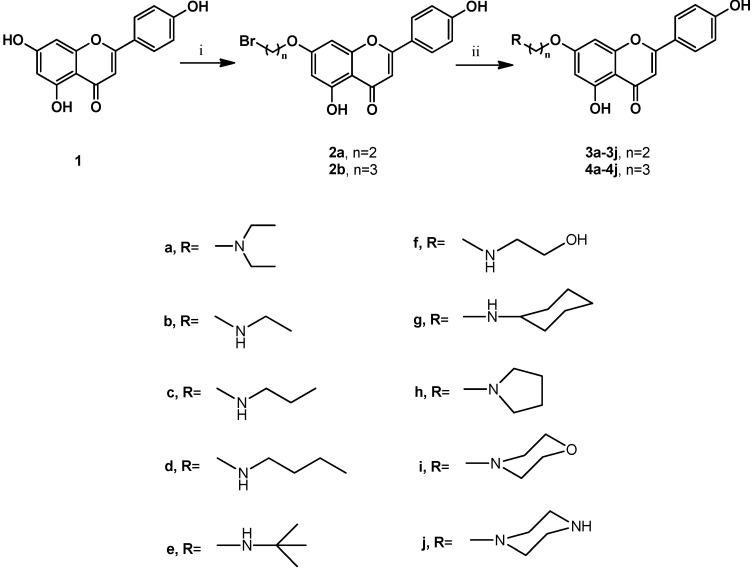
Synthesis of C-7-modified apigenin derivatives.

The synthesis of compounds **3a**–**3j** and **4a**–**4j** was accomplished according to the general pathway illustrated in Scheme 1. Compounds **2a** and **2b** were the key intermediates for the synthesis of the target compounds. They were prepared from alkylation of 7-hydroxy group using excessive amounts of 1,2-dibromoethane or 1,3-dibromopropane in the presence of potassium carbonate (K_2_CO_3_) as base in anhydrous *N,N*-dimethylformamide (DMF) at 120 °C for 2 h [17,18,19,20]. To increase the biological activities of apigenin, we synthesized apigenin derivatives, in which the apigenin ring system was linked to the alkyl amines moieties by different spacers at C-7 position to enhance their lipophilicity. Reaction of **2a** and **2b** with different cyclic and non-cyclic alkyl amines in anhydrous DMF at 80 °C for 2 h to 4 h yielded **3a**–**3j** and **4a**–**4j**, respectively. Compounds in series 1 (**3a**–**3j**) contained a 2-carbon spacer between apigenin and the substituent moieties, whereas compounds in series 2 (**4a**–**4j**) contained a 3-carbon spacer. These compounds were designated to test the importance of the substitution, together with the size of the connected groups. All synthetic compounds gave satisfactory analytical and spectroscopic data, which were in full accordance with their depicted structures.

*Reagents and conditions*: (i) BrCH_2_CH_2_Br or BrCH_2_CH_2_CH_2_Br, K_2_CO_3_, DMF, 120 °C, 2 h; (ii) R-amines, DMF, 80 °C, 2 h to 4 h.

### 2.2. Antibacterial Activity

All synthesized compounds were preliminary screened for their *in vitro* antibacterial activity against two Gram-positive bacteria [*Staphylococcus aureus* (ATCC 25923) and *Bacillus subtilis* (ATCC 6633)] and two Gram-negative bacteria [*Escherichia coli* (ATCC 25922) and *Pseudomonas aeruginosa* (ATCC 27853)] through disk diffusion method [21]. Ampicillin and tetracycline were used as positive controls [22], and dimethylsulphoxide (DMSO)-poured disk was used as negative control. Most of the synthesized compounds displayed moderate to excellent antibacterial activities against the four bacteria used at a dose of 1,000 μg/mL (Table 1). Compounds showing inhibition zones of at least 10 mm were considered active and were further evaluated for their minimum inhibitory concentrations (MICs) [23].

**Table 1:** Inhibition zones (IZ, in millimeters) (mean ^a^ ± SD ^b^) of the synthesized apigenin derivatives.

*Compounds*	*S. aureus*	*B. subtilis*	*E. coli*	*P. aeruginosa*
	*ATCC 25923*	*ATCC 6633*	*ATCC 25922*	*ATCC 27853*
**Apigenin (1)**	11.1 ± 0.3	11.0 ± 1.0	10.1 ± 0.3	10.3 ± 0.2
**2a **	11.8 ± 0.2	11.2 ± 0.3	10.2 ± 0.1	10.5 ± 0.2
**2b**	11.5 ± 0.1	11.3 ± 0.5	11.8 ± 0.3	11.9 ± 0.2
**3a **	17.0 ± 0.9	17.5 ± 0.4	12.5 ± 0.2	14.0 ± 0.3
**3b**	17.3 ± 0.3	16.5 ± 0.5	15.5 ± 0.4	14.8 ± 0.4
**3c **	17.1 ± 0.5	17.5 ± 0.3	17.0 ± 0.3	19.5 ± 0.5
**3d**	17.3 ± 0.1	17.5 ± 0.2	16.0 ± 0.3	17.5 ± 0.3
**3e**	17.5 ± 0.7	19.0 ± 0.8	16.5 ± 0.5	15.5 ± 0.2
**3f **	17.2 ± 0.4	17.5 ± 0.5	14.5 ± 0.4	15.0 ± 0.3
**3g **	21.0 ± 0.2	21.5 ± 0.7	15.5 ± 0.4	17.5 ± 0.4
**3h**	21.4 ± 0.8	21.6 ± 0.9	15.0 ± 0.6	17.2 ± 0.5
**3i**	21.7 ± 1.0	21.8 ± 1.0	19.0 ± 0.6	18.5 ± 0.6
**3j**	21.2 ± 0.5	21.0 ± 0.6	16.5 ± 0.2	17.5 ± 0.4
**4a **	21.0 ± 0.4	20.0 ± 0.4	15.0 ± 0.3	15.0 ± 0.3
**4b**	18.5 ± 0.3	19.0 ± 0.4	16.0 ± 0.1	17.5 ± 0.5
**4c **	21.1 ± 0.6	21.8 ± 0.5	18.0 ± 0.4	19.0 ± 0.6
**4d**	22.0 ± 0.8	20.0 ± 0.3	18.0 ± 0.3	21.0 ± 0.8
**4e**	18.1 ± 0.2	21.0 ± 0.2	17.5 ± 0.3	15.5 ± 0.7
**4f **	21.0 ± 0.7	17.5 ± 0.3	18.0 ± 0.5	17.5 ± 0.5
**4g **	21.9 ± 0.9	18.0 ± 0.2	18.5 ± 0.6	18.7 ± 0.9
**4h**	21.6 ± 1.0	19.0 ± 0.5	17.0 ± 0.4	17.6 ± 0.6
**4i**	24.8 ± 1.0	22.0 ± 0.7	21.0 ± 0.3	21.3 ± 1.0
**4j**	24.5 ± 0.8	21.5 ± 0.6	17.5 ± 0.3	18.0 ± 0.9
**Ampicillin**	37.3 ± 0.6	36.7 ± 1.5	28.9 ± 0.1	33.7 ± 0.5
**Tetracycline**	26.3 ± 0.5	21.0 ± 1.0	21.7 ± 1.1	24.3 ± 1.1

^a^ Mean value of measured diameters of IZs; ^b^ SD denotes standard deviation.

The MIC values are listed in Table 2. All synthesized apigenin derivatives showed higher antibacterial activities than the parent apigenin. They exhibited relatively better inhibition of Gram-positive bacteria than Gram-negative bacteria, with MIC values from 1.95 μg/mL to 15.63 μg/mL. Among **3a**–**3j**, which contained a 2-carbon spacer, **3g**–**3j** with N-heterocyclic rings at C-7 position displayed a potent zone of antibacterial activities ranging from 3.91 μg/mL to 15.63 μg/mL. In addition, **3i** exhibited prominent antibacterial activity with MICs of 3.91, 3.91, 7.81, and 7.81 μg/mL against *S. aureus*, *B. subtilis*, *E. coli*, and *P. aeruginosa*, respectively. These results were close to the antibacterial property of broad-spectrum, antibiotic tetracycline, with corresponding MICs of 1.95, 3.91, 3.91, and 3.91 μg/mL, respectively. Other 2-carbon spacer compounds (**3a**–**3f**), with aliphatic chain substituents, showed lower inhibitory activity than **3g**–**3j**. This result suggested that compounds with heterocyclic moieties at C-7 position of apigenin had much higher activity than compounds with aliphatic chains. Compounds **4a**–**4j** exhibited similar results. The N-heterocyclic rings displaced **4g**–**4j**, which showed better antibacterial activity than other compounds, namely, **4a**–**4f**, in 3-carbon spacer apigenin derivatives. Additionally, **4i** presented remarkable inhibitory activity with MIC values of 1.95, 3.91, 3.91, and 3.91 μg/mL against *S. aureus*, *B. subtilis*, *E. coli*, and *P. aeruginosa*, respectively, which is comparable with the positive control, tetracycline (1.95, 3.91, 3.91, and 3.91 μg/mL), although lower than ampicillin (0.06, 0.12, 0.98, and 0.49 μg/mL) [22]. Compounds **4a**–**4j**, which contained a 3-carbon spacer, showed higher antibacterial activity than **3a**–**3j**. This phenomenon may have been caused by the superior lipophilicity of **4a**–**4j** to **3a**–**3j**, which may have increased cell membrane permeability. The biological activity of a particular substance depends on a complex sum of individual properties including compound structure, affinity for the target site, and survival in the medium of application, survival within the biological system, transport properties, and state of the target organism [17]. Further structure-activity relationships (SARs) studies on this class of antibacterial compounds are currently under active investigation and will be reported in due course.

**Table 2:** MIC values of the synthesized apigenin derivatives against four bacterial strains.

*Compounds*	*MIC (μg/mL) ^a^*			
	*S. aureus* *ATCC 25923*	*B. subtilis* *ATCC 6633*	*E. coli* *ATCC 25922*	*P. aeruginosa* *ATCC 27853*
**Apigenin (1)**	31.25	31.25	62.5	62.5
**2a**	31.25	31.25	62.5	62.5
**2b**	31.25	31.25	31.25	31.25
**3a**	7.81	7.81	31.25	15.63
**3b**	7.81	15.63	15.63	15.63
**3c**	7.81	7.81	15.63	7.81
**3d**	7.81	7.81	15.63	7.81
**3e**	7.81	7.81	15.63	15.63
**3f**	7.81	7.81	15.63	15.63
**3g**	3.91	3.91	15.63	7.81
**3h**	3.91	3.91	15.63	7.81
**3i**	3.91	3.91	7.81	7.81
**3j**	3.91	3.91	15.63	7.81
**4a**	3.91	7.81	15.63	15.63
**4b**	7.81	7.81	15.63	7.81
**4c**	3.91	3.91	7.81	7.81
**4d**	3.91	3.91	7.81	3.91
**4e**	7.81	3.91	7.81	15.63
**4f**	3.91	7.81	7.81	7.81
**4g**	3.91	7.81	7.81	7.81
**4h**	3.91	7.81	7.81	7.81
**4i**	1.95	3.91	3.91	3.91
**4j**	1.95	3.91	7.81	7.81
**Ampicillin**	0.06	0.12	0.98	0.49
**Tetracycline**	1.95	3.91	3.91	3.91

^a^ Average of three parallel experiments.

### 2.3. Antiproliferative Activity

The inhibitory effects of all the synthesized apigenin derivatives, **3a**–**3j** and **4a**–**4j**, were assayed by evaluating cell proliferation on A549, HeLa, HepG2, and MCF-7 cell lines. The results are summarized in Table 3.

**Table 3:** Antiproliferative activities (IC_50_) of the synthesized apigenin derivatives against A549, HeLa, HepG2, and MCF-7 cell lines.

Compounds	IC_50_ (μg/mL)			
	A549	HeLa	HepG2	MCF-7
**Apigenin (1)**	1,740 ± 3.4	450 ± 2.0	460 ± 2.2	>2,000 ± 3.6
**2a**	643 ± 4.2	590 ± 4.1	408 ± 3.8	534 ± 3.4
**2b**	251 ± 3.4	233 ± 2.2	176 ± 2.1	209 ± 2.8
**3a**	101 ± 3.0	110 ± 2.0	116 ± 2.3	125 ± 2.2
**3b**	94 ± 2.5	109 ± 1.9	103 ± 1.9	102 ± 2.1
**3c**	87 ± 2.2	98 ± 1.5	105 ± 1.4	114 ± 1.9
**3d**	80 ± 1.5	117 ± 2.1	112 ± 2.0	137 ± 2.6
**3e**	78 ± 1.6	97 ± 1.8	92 ± 1.8	99 ± 2.0
**3f**	83 ± 2.3	89 ± 1.4	96 ± 1.5	92 ± 2.0
**3g**	42 ± 2.1	32 ± 1.2	33 ± 1.2	71 ± 1.2
**3h**	45 ± 1.7	46 ± 1.0	43 ± 1.7	70 ± 1.8
**3i**	39 ± 0.9	42 ± 0.9	36 ± 1.0	54 ± 1.1
**3j**	26 ± 1.0	17 ± 0.8	29 ± 1.2	49 ± 1.4
**4a**	91 ± 2.4	99 ± 2.3	100 ± 2.8	99 ± 2.1
**4b**	85 ± 2.1	83 ± 1.7	94 ± 2.2	87 ± 2.3
**4c**	72 ± 1.8	81 ± 1.3	82 ± 1.8	95 ± 2.8
**4d**	63 ± 1.1	101 ± 2.6	83 ± 1.7	103 ± 3.1
**4e**	62 ± 1.5	86 ± 2.0	90 ± 2.0	88 ± 2.9
**4f**	70 ± 2.2	75 ± 1.3	89 ± 1.4	89 ± 3.3
**4g**	32 ± 1.4	27 ± 1.1	31 ± 1.0	59 ± 2.0
**4h**	35 ± 2.0	34 ± 1.5	44 ± 1.2	50 ± 2.3
**4i**	27 ± 1.3	19 ± 1.0	28 ± 1.1	47 ± 1.9
**4j**	16 ± 1.0	11 ± 1.1	25 ± 1.6	32 ± 1.8

All the synthesized derivatives, **3a**–**3j** and **4a**–**4j**, showed relatively higher antiproliferative activity compared with the parent apigenin. Compounds **3a**–**3j**, which contained a 2-carbon spacer, had moderate activities against the four selected cancer cell lines. In this series, **3g**–**3j**, with half maximal inhibitory concentration (IC_50_) ranging from 17 μg/mL to 71 μg/mL, showed better activities than **3a**–**3f**. Notable effect was exerted by **3j**, which had the lowest IC_50_ values against the four cancer cells, especially against HeLa cells (IC_50_ = 17 μg/mL). Similarly, in **4a**–**4j**, **4g**–**4j** exhibited stronger activities than **4a**–**4f**. Compound **4j** showed notable activity against the four cancer cell lines. As shown in Table 3, compared with the compounds with 2-carbon spacer, 3-carbon-spacer-containing compounds **4a**–**4j**, showed a slight increase in antiproliferative activity against the four cancer cell lines. This result may have been caused by the elongation of the alkyl side chain from two to three C-atoms that retained the modulating activity, with C_3_-bridge derivatives being the most active [15]. The compounds containing N-heterocyclic-ring amino side chains (**3g**–**3j** and **4g**–**4j**) displayed better activities against the four cancer cell lines than those containing alkyl amino side chains. As reference [15] described, this result may be attributed to the increasing lipophilicity of these compounds, which could influence their ability to reach the target via transmembrane diffusion. In most cases, the presence or introduction of various functional groups in a compound does not allow to accurately explain the type and intensity of its biological activity [15]. So further studies on the mechanism of action will be in the succeeding work.

## 3. Experimental

### 3.1. General

Reagents and solvents were purchased commercially and used without further purification, unless otherwise stated. Reactions were monitored by thin layer chromatography, performed on silica gel glass plates (GF_254_, Qingdao Haiyang Chemical Co., Ltd., Shandong, China). Plates were visualized under UV light using ZF-6 type III UV analyzer (Shanghai Jiapeng Technology Co., Ltd, Shanghai, China). Column chromatography was performed on silica gel (200–300 mesh) to purify the compounds. Melting points were measured using a digital melting point apparatus (Zhengzhou Mingze Technology Co., Ltd, Zhengzhou, China) and were uncorrected. The NMR spectra were obtained using a Bruker Avance DMX 500 MHz or 400 MHz instrument (Bruker, Billerica, MA, USA). ^1^H-NMR (500 or 400 MHz) and ^13^C-NMR (125 or 100 MHz) were run in deuterated DMSO (DMSO-*d_6_*). Chemical shifts were related to those of the solvent. Mass spectra (MS) were acquired using electrospray ionization (ESI) technique on a Thermo Scientific LCQ FLEET mass spectrometer (Thermo Fisher Scientific, Waltham, MA, USA). MIC was detected using BIO-RAD 680 microplate reader (Beijing Yuanye Bio Co., Ltd, Beijing, China). Deionized water (Milli-Q, Millipore, Bedford, MA, USA) was used to prepare aqueous solutions. Primary and secondary amines were obtained from Aladdin (Shanghai, China). DMF was purified by distillation under vacuum over CaH_2_ before utilization. Apigenin (>98%) was purchased from Xi’an Spring-chem Bio-tech Co., Ltd. (Xi’an, Shaanxi, China) and was used without further purification. Ampicillin and MTT were purchased from Amersco Inc. (Solon, OH, USA). Tetracycline was acquired from Sigma-Aldrich (St. Louis, MO, USA). Yeast extract and tryptone were obtained from Oxoid Ltd. (Basingstoke, Hampshire, UK).

*Apigenin* (**1**). Yellow powder, m.p. 347–348 °C; ^1^H-NMR (500 MHz, DMSO-*d_6_*) δ: 6.20 (1H, d, *J* = 2.0 Hz, H-6), 6.48 (1H, d, *J* = 2.0 Hz, H-8), 6.79 (1H, s, H-3), 6.93 (2H, d, *J* = 10.0 Hz, H-3′ and H-5′), 7.93 (2H, d, *J* = 10.0 Hz, H-2′ and H-6′), 10.35 (1H, s, 4′-OH), 10.82 (1H, s, 7-OH), 12.97 (1H, s, 5-OH). ^13^C-NMR (125 MHz, DMSO-*d_6_*) δ: 94.87, 99.74, 103.76, 104.61, 116.87, 122.09, 129.39, 158.22, 162.08, 162.37, 164.65, 165.04, 182.66. ESI-MS (−) for C_15_H_10_O_5_: [M-H]^–^_(found)_ = 269.0 [M-H]^–^_(calcd)_ = 269.0.

### 3.2. Synthesis of Compounds **2a**, **2b**, **3a**–**3j**, and **4a**–**4j**

*7-(2-Bromoethoxy)-5-hydroxy-2-(4-hydroxyphenyl)-4H-chromen-4-one* (**2a**). To a solution of **1** (1.35 g, 5 mmol) in 100 mL of anhydrous DMF, 1,2-dibromoethane (23.5 g, 125 mmol) and anhydrous potassium carbonate (0.7 g, 5 mmol) were added, followed by heating at 120 °C for 2 h. After the completion of reaction, the resulting mixture was cooled to room temperature and filtered. The filtrate was concentrated, and the residue was purified by column chromatography (petroleum ether/ethyl acetate = 2:1) to obtain **2a** in 70% yield, pale yellow needle, m.p. 226–228 °C; ^1^H-NMR (500 MHz, DMSO-*d_6_*) δ: 3.86 (t, 2H, OCH_2_CH_2_, *J* = 5.3 Hz), 4.47 (t, 2H, OCH_2_CH_2_, *J* = 5.3 Hz), 6.42 (d, 1H, 6-H, *J* = 2.1 Hz), 6.84 (d, 1H, 8-H, *J* = 2.1 Hz), 6.88 (s, 1H, 3-H), 6.95 (d, 2H, 3′,5′-2H, *J* = 8.8 Hz), 7.98 (d, 2H, 2′,6′-2H, *J* = 8.8 Hz), 10.41 (s, 1H, 4’-OH), 12.99 (s, 1H, 5-OH). ^13^C-NMR (125 MHz, DMSO-*d_6_*) δ: 31.37, 68.91, 93.76, 98.83, 103.49, 105.39, 116.45, 121.48, 129.05, 157.64, 161.70, 161.79, 164.09, 164.62, 182.39. ESI-MS (−) for C_17_H_13_BrO_5_: [M-H]^–^_(found)_ = 376.0 [M-H]^–^_(calcd)_ = 376.0.

*7-(3-Bromopropoxy)-5-hydroxy-2-(4-hydroxyphenyl)-4H-chromen-4-one* (**2b**). To a solution of **1** (1.35 g, 5 mmol) in 100 mL of anhydrous DMF, 1,3-dibromopropane (25.5 g, 125 mmol) and anhydrous potassium carbonate (0.7 g, 5 mmol) were added, followed by heating at 120 °C for 2 h. After the completion of reaction, the resulting mixture was cooled to room temperature and filtered. The filtrate was concentrated and the residue was purified using column chromatography (petroleum ether/ethyl acetate = 2:1) to obtain **2b** in 68% yield, pale yellow needle, m.p. 204–205 °C; ^1^H-NMR (500 MHz, DMSO-*d_6_*) δ: 2.29 (m, 2H, OCH_2_CH_2_CH_2_), 3.69 (t, 2H, OCH_2_CH_2_CH_2_, *J* = 5.0 Hz), 4.21 (s, 2H, OCH_2_CH_2_CH_2_), 6.38 (s, 1H, 6-H), 6.80 (s, 1H, 8-H), 6.85 (s, 1H, 3-H), 6.95 (d, 2H, 3′,5′-2H, *J* = 7.9 Hz), 7.96 (d, 2H, 2′,6′-2H, *J* = 7.9 Hz), 12.97 (s, 1H, 5-OH). ^13^C-NMR (125 MHz, DMSO-*d_6_*) δ: 31.47, 32.01, 66.70, 93.53, 98.81, 103.39, 105.24, 116.50, 121.29, 129.02, 157.68, 161.66, 162.09, 164.58, 164.66, 182.37. ESI-MS (−) for C_18_H_15_BrO_5_: [M-H]^–^_(found)_ = 390.1 [M-H]^–^_(calcd)_ = 390.0.

*7-[2-(Diethylamino)ethoxy]-5-hydroxy-2-(4-hydroxyphenyl)-4H-chromen-4-one* (**3a**). To a solution of **2a** (0.37 g, 1 mmol) in 30 mL of anhydrous DMF, diethylamine (0.73 g, 10 mmol) was added, followed by heating at 80 °C for 3 h. The solvent was evaporated, and the residue was purified using column chromatography to obtain **3a** in 70% yield, pale yellow powder, m.p. 237–238 °C; ^1^H-NMR (500 MHz, DMSO-*d_6_*) δ: 0.98 (t, 6H, N(CH_2_CH_3_)_2_, *J* = 7.0 Hz), 2.56 (q, 4H, N(CH_2_CH_3_)_2_, *J* = 7.0 Hz), 2.80 (t, 2H, CH_2_, *J* = 5.4 Hz), 4.13 (t, 2H, CH_2_, *J* = 5.4 Hz), 6.33 (s, 1H, 6-H), 6.76 (s, 1H, 8-H), 6.81 (s, 1H, 3-H), 6.93 (d, 2H, 3′,5′-2H, *J* = 8.4 Hz), 7.94 (d, 2H, 2′,6′-2H, *J* = 8.4 Hz). ^13^C-NMR (125 MHz, DMSO-*d_6_*) δ: 12.27, 47.38, 51.49, 67.66, 93.51, 98.74, 103.28, 105.06, 116.52, 121.23, 128.98, 157.65, 161.61, 162.14, 164.55, 164.82, 182.31. ESI-MS (-) for C_21_H_23_NO_5_: [M-H]^–^_(found)_ = 368.2 [M-H]^–^_(calcd)_ = 368.1.

*7-[2-(Ethylamino)ethoxy]-5-hydroxy-2-(4-hydroxyphenyl)-4H-chromen-4-one* (**3b**). Prepared as described for **3a**, with **2a** (0.37 g, 1 mmol) and ethylamine (0.45 g, 10 mmol). Yield 67%, pale yellow powder, m.p. 202–203 °C; ^1^H-NMR (500 MHz, DMSO-*d_6_*) δ: 1.23 (t, 3H, NCH_2_CH_3_, *J* = 7.2 Hz), 2.99 (q, 2H, NCH_2_CH_3_, *J* = 7.2 Hz), 3.31 (t, 2H, CH_2_, *J* = 4.9 Hz), 4.36 (t, 2H, CH_2_, *J* = 4.9 Hz), 6.41 (s, 1H, 6-H), 6.80 (s, 1H, 8-H), 6.83 (s, 1H, 3-H), 6.95 (d, 2H, 3′,5′-2H, *J* = 8.8 Hz), 7.94 (d, 2H, 2′,6′-2H, *J* = 8.8 Hz). ^13^C-NMR (125 MHz, DMSO-*d_6_*) δ: 11.93, 43.08, 46.02, 65.13, 93.61, 98.96, 103.51, 105.51, 116.45, 121.40, 128.91, 157.62, 161.69, 161.85, 163.92, 164.67, 182.35. ESI-MS (−) for C_19_H_19_NO_5_: [M-H]^–^_(found)_ = 340.0 [M-H]^–^_(calcd)_ = 340.1.

*7-[2-(Propylamino)ethoxy]-5-hydroxy-2-(4-hydroxyphenyl)-4H-chromen-4-one* (**3c**). Prepared as described for **3a**, with **2a** (0.37 g, 1 mmol) and propylamine (0.59 g, 10 mmol). Yield 75%, pale yellow powder, m.p. 242–244 °C; ^1^H-NMR (500 MHz, DMSO-*d_6_*) δ: 0.94 (t, 3H, NCH_2_CH_2_CH_3_, *J* = 7.4 Hz), 1.63–1.68 (m, 2H, NCH_2_CH_2_CH_3_), 2.96 (t, 2H, NCH_2_CH_2_CH_3_, *J* = 7.4 Hz), 3.37 (t, 2 H, CH_2_, *J* = 5.0 Hz), 4.38 (t, 2H, CH_2_, *J* = 5.0 Hz), 6.45 (d, 1H, 6-H, *J* = 2.1 Hz), 6.86 (d, 1H, 8-H, *J* = 2.1 Hz), 6.90 (s, 1H, 3-H), 6.96 (d, 2H, 3′,5′-2H, *J* = 8.8 Hz), 7.99 (d, 2H, 2′,6′-2H, *J* = 8.8 Hz), 12.92 (s, 1H, 5-OH). ^13^C-NMR (125 MHz, DMSO-*d_6_*) δ: 11.47, 19.72, 46.26, 49.41, 64.99, 93.77, 99.01, 103.59, 105.51, 116.49, 121.44, 129.10, 157.64, 161.66, 161.88, 163.93, 164.69, 182.44. ESI-MS (−) for C_20_H_21_NO_5_: [M-H]^–^_(found)_ = 354.1 [M-H]^–^_(calcd)_ = 354.3.

*7-[2-(n-Butylamino)ethoxy]-5-hydroxy-2-(4-hydroxyphenyl)-4H-chromen-4-one* (**3d**). Prepared as described for **3a**, with **2a** (0.37 g, 1 mmol) and *n*-butylamine (0.73 g, 10 mmol). Yield 73%, pale yellow powder, m.p. 247–248 °C; ^1^H-NMR (500 MHz, DMSO-*d_6_*) δ: 0.92 (t, 3H, NCH_2_CH_2_CH_2_CH_3_, *J* = 7.4 Hz), 1.34–1.38 (m, 4H, NCH_2_CH_2_CH_2_CH_3_), 1.58 (t, 2H, NCH_2_CH_2_CH_2_CH_3_, *J* = 7.4 Hz), 2.91 (t, 2H, CH_2_, *J* = 7.5 Hz), 4.34 (t, 2H, CH_2_, *J* = 7.5 Hz), 6.44 (d, 1H, 6-H, *J* = 2.0 Hz), 6.85 (d, 1H, 8-H, *J* = 2.0 Hz), 6.90 (s, 1H, 3-H), 6.96 (d, 2H, 3′,5′-2H, *J* = 8.7 Hz), 7.99 (d, 2H, 2′,6′-2H, *J* = 8.7 Hz), 12.91 (s, 1H, 5-OH). ^13^C-NMR (125 MHz, DMSO-*d_6_*) δ: 14.08, 19.92, 28.89, 46.62, 47.87, 65.64, 93.73, 98.99, 103.56, 105.43, 116.49, 121.45, 129.07, 157.65, 161.64, 161.88, 164.11, 164.67, 182.41. ESI-MS (−) for C_21_H_23_NO_5_: [M-H]^–^_(found)_ = 368.1 [M-H]^–^_(calcd)_ = 368.1.

*7-[2-(tert-Butylamino)ethoxy]-5-hydroxy-2-(4-hydroxyphenyl)-4H-chromen-4-one* (**3e**). Prepared as described for **3a**, with **2a** (0.37 g, 1 mmol) and *tert*-butylamine (0.73 g, 10 mmol). Yield 71%, pale yellow powder, m.p. 263–265 °C; ^1^H-NMR (500 MHz, DMSO-*d_6_*) δ: 1.34(s, 9H, (CH_3_)_3_), 2.91 (t, 2H, CH_2_, *J* = 5.0 Hz), 4.38 (t, 2H, CH_2_, *J* = 5.0 Hz), 6.46 (d, 1H, 6-H, *J* = 2.0 Hz), 6.87 (d, 1H, 8-H, *J* = 2.0 Hz), 6.91 (s, 1H, 3-H), 6.96 (d, 2H, 3′,5′-2H, *J* = 8.7 Hz), 8.00 (d, 2H, 2′,6′-2H, *J* = 8.7 Hz), 13.03 (s, 1H, 5-OH). ^13^C-NMR (125 MHz, DMSO-*d_6_*) δ: 25.64, 27.61, 51.61, 65.26, 93.81, 99.01, 103.58, 105.53, 116.49, 121.43, 129.10, 157.64, 161.66, 161.89, 163.94, 164.72, 182.43. ESI-MS (−) for C_21_H_23_NO_5_: [M-H]^–^_(found)_ = 368.1 [M-H]^–^_(calcd)_ = 368.1.

*7-[2-(2′-Hydroxyethylamino)ethoxy]-5-hydroxy-2-(4-hydroxyphenyl)-4H-chromen-4-one* (**3f**). Prepared as described for **3a**, with **2a** (0.37 g, 1 mmol) and ethanolamine (0.61 g, 10 mmol). Yield 73%, pale yellow powder, m.p. 224–226 °C; ^1^H-NMR (500 MHz, DMSO-d_6_) δ: 3.10 (s, 2H, NCH_2_CH_2_OH), 3.38 (s, 2H, NCH_2_CH_2_OH), 3.70 (s, 2H, CH_2_), 4.41 (s, 2H, CH_2_), 6.44 (s, 1H, 6-H), 6.86 (s, 1H, 8-H), 6.89 (s, 1H, 3-H), 6.96 (d, 2H, 3′,5′-2H, *J* = 8.5 Hz), 7.98 (d, 2H, 2′,6′-2H, *J* = 8.5 Hz), 12.97 (s, 1H, 5-OH). ^13^C-NMR (125 MHz, DMSO-*d_6_*) δ: 46.28, 49.90, 57.08, 64.97, 93.78, 99.03, 103.48, 105.49, 116.49, 121.44, 129.08, 157.65, 161.65, 161.89, 163.96, 164.68, 182.43. ESI-MS (−) for C_19_H_19_NO_6_: [M-H]^–^_(found)_ = 356.1 [M-H]^–^_(calcd)_ = 356.1.

*7-[2-(Cyclohexylamino)ethoxy]-5-hydroxy-2-(4-hydroxyphenyl)-4H-chromen-4-one* (**3g**). Prepared as described for **3a**, with **2a** (0.37 g, 1 mmol) and cyclohexylamine (0.99 g, 10 mmol). Yield 68%, pale yellow powder, m.p. 269–270 °C; ^1^H-NMR (500 MHz, DMSO-*d_6_*) δ: 1.13 (d, 2H, CH_2_, *J* = 10.0 Hz), 1.26–1.29 (m, 6H), 1.63 (d, 2H, CH_2_, *J* = 10.0 Hz), 2.05 (s, 2H, CH_2_), 3.00 (d, 2H, CH_2_, *J* = 5.0 Hz), 4.35 (d, 2H, CH_2_, *J* = 4.9 Hz), 6.44 (d, 1H, 6-H, *J* = 2.0 Hz), 6.86 (d, 1H, 8-H, *J* = 2.0 Hz), 6.90 (s, 1H, 3-H), 6.96 (d, 2H, 3′,5′-2H, *J* = 8.8 Hz), 7.99 (d, 2H, 2′,6′-2H, *J* = 8.8 Hz), 12.92 (s, 1H, 5-OH). ^13^C-NMR (125 MHz, DMSO-*d_6_*) δ: 24.49, 25.37, 30.84, 43.55, 49.79, 56.88, 93.76, 98.97, 103.60, 105.49, 116.48, 121.45, 129.10, 157.65, 161.67, 161.88, 164.04, 164.68, 182.44. ESI-MS (−) for C_23_H_25_NO_5_: [M-H]^–^_(found)_ = 394.1 [M-H]^–^_(calcd)_ = 394.2.

*7-[2-(Pyrrolidin-1-yl)ethoxy]-5-hydroxy-2-(4-hydroxyphenyl)-4H-chromen-4-one* (**3h**). Prepared as described for **3a**, with **2a** (0.37 g, 1 mmol) and pyrrolidine (0.71 g, 10 mmol). Yield 70%, pale yellow powder, m.p. 252–254 °C; ^1^H-NMR (500 MHz, DMSO-*d_6_*) δ: 1.90 (br s, 2H, CH_2_), 2.01 (s, 4H), 3.14 (s, 2H, CH_2_), 3.64 (s, 2H, CH_2_), 4.46 (s, 2H, CH_2_), 6.44 (d, 1H, 6-H, *J* = 1.8 Hz), 6.78 (d, 2H, 8-H, 3-H, *J* = 1.8 Hz), 6.94 (d, 2H, 3′,5′-2H, *J* = 8.8 Hz), 7.90 (d, 2H, 2′,6′-2H, *J* = 8.8 Hz), 10.27 (s, 1H, 4’-OH), 12.98 (s, 1H, 5-OH). ^13^C-NMR (125 MHz, DMSO-*d_6_*) δ: 22.63, 44.89, 52.77, 54.03, 93.19, 98.40, 103.11, 105.22, 115.95, 120.97, 128.30, 157.15, 161.39, 161.46, 163.09, 164.24, 181.88. ESI-MS (−) for C_21_H_21_NO_5_: [M-H]^–^_(found)_ = 366.1 [M-H]^–^_(calcd)_ = 366.1.

*7-[2-(Morpholin-4-yl)ethoxy]-5-hydroxy-2-(4-hydroxyphenyl)-4H-chromen-4-one* (**3i**). Prepared as described for **3a**, with **2a** (0.37 g, 1 mmol) and morpholine (0.87 g, 10 mmol). Yield 75%, pale yellow powder, m.p. 199–200 °C; ^1^H-NMR (500 MHz, DMSO-d_6_) δ: 2.74 (s, 4H), 2.90 (s, 2H, CH_2_), 3.59 (d, 4H, *J* = 5.6 Hz), 4.22 (t, 2H, CH_2_, *J* = 5.6 Hz), 6.39 (s, 1H, 6-H), 6.81 (s, 1H, 8-H), 6.86 (s, 1H, 3-H), 6.95 (d, 2H, 3′,5′-2H, *J* = 8.5 Hz), 7.97 (d, 2H, 2′,6′-2H, *J* = 8.5 Hz), 10.41 (s, 1H, 4’-OH), 12.96 (s, 1H, 5-OH). ^13^C-NMR (125 MHz, DMSO-*d_6_*) δ: 45.58, 54.02, 57.14, 66.64, 93.67, 98.84, 103.49, 105.15, 116.45, 121.55, 129.02, 157.67, 161.79, 162.78, 163.76, 164.76, 182.38. ESI-MS (−) for C_21_H_21_NO_6_: [M-H]^–^_(found)_ = 382.1 [M-H]^–^_(calcd)_ = 382.1.

*7-[2-(Piperazin-1-yl)ethoxy]-5-hydroxy-2-(4-hydroxyphenyl)-4H-chromen-4-one* (**3j**). Prepared as described for **3a**, with **2a** (0.37 g, 1 mmol) and piperazine (0.86 g, 10 mmol). Yield 65%, pale yellow powder, m.p. 229–230 °C; ^1^H-NMR (500 MHz, DMSO-*d_6_*) δ: 2.81 (s, 2H, CH_2_), 2.84–2.86 (m, 1H, NH), 2.91 (d, 2H, CH_2_, *J* = 5.3 Hz), 3.09 (s, 4H), 3.46 (d, 2H, CH_2_, *J* = 4.3 Hz), 4.23 (s, 2H, CH_2_), 6.39 (s, 1H, 6-H), 6.81 (s, 1H, 8-H), 6.87 (s, 1H, 3-H), 6.96 (d, 2H, 3′,5′-2H, *J* = 8.6 Hz), 7.97 (d, 2H, 2′,6′-2H, *J* = 8.6 Hz), 12.95 (s, 1H, 5-OH). ^13^C-NMR (125 MHz, DMSO-*d_6_*) δ: 43.49, 50.13, 56.32, 66.81, 93.71, 98.86, 103.49, 105.18, 116.48, 121.47, 129.02, 157.67, 161.49, 161.65, 164.56, 164.64, 182.38. ESI-MS (−) for C_21_H_22_N_2_O_5_: [M-H]^–^_(found)_ = 381.1 [M-H]^–^_(calcd)_ = 381.1.

*7-[3-(Diethylamino)propoxy]-5-hydroxy-2-(4-hydroxyphenyl)-4H-chromen-4-one* (**4a**). Prepared as described for **3a**, with **2b** (0.39 g, 1 mmol) and diethylamino (0.73 g, 10 mmol). Yield 73%, pale yellow powder, m.p. 227–229 °C; ^1^H-NMR (500 MHz, DMSO-*d_6_*) δ: 1.24 (t, 6H, N(CH_2_CH_3_)_2_, *J* = 7.1 Hz), 2.15 (s, 2H, CH_2_), 3.17–3.22 (m, 6H), 4.21 (t, 2H, CH_2_, *J* = 5.8 Hz), 6.39 (s, 1H, 6-H), 6.79 (s, 1H, 8-H), 6.86 (s, 1H, 3-H), 6.96 (d, 2H, 3′,5′-2H, *J* = 8.6 Hz), 7.97 (d, 2H, 2′,6′-2H, *J* = 8.6 Hz), 10.42 (s, 1H, 4’-OH), 12.98 (s, 1H, 5-OH). ^13^C-NMR (125 MHz, DMSO-*d_6_*) δ: 9.16, 23.55, 46.93, 48.28, 66.13, 93.67, 98.77, 103.51, 105.25, 116.46, 121.47, 129.02, 157.63, 161.69, 161.83, 164.45, 164.58, 182.38. ESI-MS (+) for C_22_H_25_NO_5_: [M+H]^+^_(found)_ = 384.2 [M+H]^+^_(calcd)_ = 384.2.

*7-[3-(Ethylamino)propoxy]-5-hydroxy-2-(4-hydroxyphenyl)-4H-chromen-4-one* (**4b**). Prepared as described for **3a**, with **2b** (0.39 g, 1 mmol) and ethylamine (0.45 g, 10 mmol). Yield 75%, pale yellow powder, m.p. 226–228 °C; ^1^H-NMR (400 MHz, DMSO-*d_6_*) δ: 1.20 (t, 3H, NCH_2_CH_3_, *J* = 7.2 Hz), 2.11 (q, 2H, NCH_2_CH_3_, *J* = 7.2 Hz), 2.95–3.01 (m, 2H, CH_2_), 3.06 (t, 2H, CH_2_, *J* = 6.0 Hz), 4.21 (t, 2H, CH_2_, *J* = 6.0 Hz), 6.39 (d, 1H, 6-H, *J* = 2.2 Hz), 6.79 (d, 1H, 8-H, *J* = 2.2 Hz), 6.87 (s, 1H, 3-H), 6.95 (d, 2H, 3′,5′-2H, *J* = 8.8 Hz), 7.96 (d, 2H, 2′,6′-2H, *J* = 8.8 Hz). ^13^C-NMR (100 MHz, DMSO-*d_6_*) δ: 11.60, 25.87, 42.58, 44.04, 66.09, 93.67, 98.81, 103.49, 105.21, 116.48, 121.45, 129.03, 157.64, 161.66, 161.84, 164.51, 164.59, 182.39. ESI-MS (+) for C_20_H_21_NO_5_: [M+H]^+^_(found)_ = 356.1 [M+H]^+^_(calcd)_ = 356.1.

*7-[3-(Propylamino)propoxy]-5-hydroxy-2-(4-hydroxyphenyl)-4H-chromen-4-one* (**4c**). Prepared as described for **3a**, with **2b** (0.39 g, 1 mmol) and propylamine (0.59 g, 10 mmol). Yield 78%, pale yellow powder, m.p. 220–221 °C; ^1^H-NMR (400 MHz, DMSO-*d_6_*) δ: 0.93 (t, 3H, NCH_2_CH_2_CH_3_, *J* = 7.4 Hz), 1.59–1.68 (m, 2H, CH_2_), 2.08–2.15 (m, 2H, CH_2_), 2.90 (t, 2H, CH_2_, *J* = 7.4 Hz), 3.08 (t, 2H, CH_2_, *J* = 6.1 Hz), 4.21 (t, 2H, CH_2_, *J* = 6.1 Hz), 6.39 (d, 1H, 6-H, *J* = 2.2 Hz), 6.78 (d, 1H, 8-H, *J* = 2.2 Hz), 6.86 (s, 1H, 3-H), 6.95 (d, 2H, 3′,5′-2H, *J* = 8.8 Hz), 7.96 (d, 2H, 2′,6′-2H, *J* = 8.8 Hz), 12.97 (s, 1H, 5-OH). ^13^C-NMR (100 MHz, DMSO-*d_6_*) δ: 11.40, 19.54, 25.73, 44.51, 48.97, 66.09, 93.68, 98.81, 103.51, 105.22, 116.48, 121.47, 129.02, 157.65, 161.66, 161.82, 164.50, 164.61, 182.38. ESI-MS (+) for C_21_H_23_NO_5_: [M+H]^+^_(found)_ = 370.1 [M+H]^+^_(calcd)_ = 370.2.

*7-[3-(n-Butylamino)propoxy]-5-hydroxy-2-(4-hydroxyphenyl)-4H-chromen-4-one* (**4d**). Prepared as described for **3a**, with **2b** (0.39 g, 1 mmol) and *n*-butylamine (0.73 g, 10 mmol). Yield 71%, pale yellow powder, m.p. 246–248 °C; ^1^H-NMR (400 MHz, DMSO-*d_6_*) δ: 0.91 (t, 3H, NCH_2_CH_2_CH_2_CH_3_, *J* = 7.3 Hz), 1.32–1.37 (m, 2H, CH_2_), 1.55–1.62 (m, 2H, CH_2_), 2.09–2.12 (m, 2H, CH_2_), 2.93 (t, 2H, CH_2_, *J* = 7.3 Hz), 3.08 (t, 2H, CH_2_, *J* = 6.0 Hz), 4.20 (t, 2H, CH_2_, *J* = 6.0 Hz), 6.38 (d, 1H, 6-H, *J* = 2.1 Hz), 6.79 (d, 1H, 8-H, *J* = 2.1 Hz), 6.86 (s, 1H, 3-H), 6.95 (d, 2H, 3′,5′-2H, *J* = 8.8 Hz), 7.96 (d, 2H, 2′,6′-2H, *J* = 8.8 Hz). ^13^C-NMR (100 MHz, DMSO-*d_6_*) δ: 13.98, 19.75, 25.79, 28.10, 44.55, 47.16, 66.09, 93.66, 98.79, 103.59, 105.21, 116.47, 121.45, 129.02, 157.63, 161.65, 161.82, 164.49, 164.58, 182.38. ESI-MS (+) for C_22_H_25_NO_5_: [M+H]^+^_(found)_ = 384.1 [M+H]^+^_(calcd)_ = 384.2.

*7-[3-(tert-Butylamino)propoxy]-5-hydroxy-2-(4-hydroxyphenyl)-4H-chromen-4-one* (**4e**). Prepared as described for **3a**, with **2b** (0.39 g, 1 mmol) and *tert*-butylamine (0.73 g, 10 mmol). Yield 69%, pale yellow powder, m.p. 163–165 °C; ^1^H-NMR (400 MHz, DMSO-*d_6_*) δ: 1.32 [s, 9H, (CH_3_)_3_], 2.14 (s, 2H, CH_2_), 3.06 (t, 2H, CH_2_, *J* = 6.1 Hz), 4.23 (t, 2H, CH_2_, *J* = 6.1 Hz), 6.40 (s, 1H, 6-H), 6.80 (s, 1H, 8-H), 6.86 (s, 1H, 3-H), 6.95 (d, 2H, 3′,5′-2H, *J* = 8.8 Hz), 7.96 (d, 2H, 2′,6′-2H, *J* = 8.8 Hz), 12.97 (s, 1H, 5-OH). ^13^C-NMR (100 MHz, DMSO-*d_6_*) δ: 25.65, 26.41, 56.80, 58.35, 66.09, 93.70, 98.78, 103.49, 105.21, 116.46, 121.46, 129.02, 157.63, 161.68, 161.82, 164.50, 164.57, 182.38. ESI-MS (+) for C_22_H_25_NO_5_: [M+H]^+^_(found)_ = 384.1 [M+H]^+^_(calcd)_ = 384.2.

*7-[3-(2′-Hydroxyethylamino)propoxy]-5-hydroxy-2-(4-hydroxyphenyl)-4H-chromen-4-one* (**4f**). Prepared as described for **3a**, with **2b** (0.39 g, 1 mmol) and ethanolamine (0.61 g, 10 mmol). Yield 67%, pale yellow powder, m.p. 244–246 °C; ^1^H-NMR (400 MHz, DMSO-*d_6_*) δ: 2.15 (t, 2H, NCH_2_CH_2_OH, *J* = 5.2 Hz), 3.05 (t, 2H, NCH_2_CH_2_OH, *J* = 5.2 Hz), 3.12–3.15 (m, 2H, CH_2_), 3.69 (s, 2H, CH_2_), 4.21 (t, 2H, CH_2_, *J* = 6.0 Hz), 6.38 (d, 1H, 6-H, *J* = 2.1 Hz), 6.79 (d, 1H, 8-H, *J* = 2.1 Hz), 6.85 (s, 1H, 3-H), 6.96 (d, 2H, 3′,5′-2H, *J* = 8.8 Hz), 7.96 (d, 2H, 2′,6′-2H, *J* = 8.8 Hz), 12.96 (s, 1H, 5-OH). ^13^C-NMR (100 MHz, DMSO-*d_6_*) δ: 44.65, 49.49, 56.81, 60.16, 66.22, 93.69, 98.83, 103.52, 105.23, 116.48, 121.48, 129.01, 157.64, 161.66, 161.84, 164.52, 164.60, 182.37. ESI-MS (+) for C_20_H_21_NO_6_: [M+H]^+^_(found)_ = 372.1 [M+H]^+^_(calcd)_ = 372.1.

*7-[3-(Cyclohexylamino)propoxy]-5-hydroxy-2-(4-hydroxyphenyl)-4H-chromen-4-one* (**4g**). Prepared as described for **3a**, with **2b** (0.39 g, 1 mmol) and cyclohexylamine (0.99 g, 10 mmol). Yield 62%, pale yellow powder, m.p. 160–162 °C; ^1^H-NMR (400 MHz, DMSO-*d_6_*) δ: 1.10–1.16 (m, 2H), 1.21–1.34 (m, 6H), 1.76–1.79 (m, 3H), 2.14 (d, 2H, *J* = 6.4 Hz), 3.10 (t, 2H, *J* = 6.1 Hz), 4.21 (t, 2H, *J* = 6.1 Hz), 6.38 (d, 1H, 6-H, *J* = 2.1 Hz), 6.78 (d, 1H, 8-H, *J* = 2.1 Hz), 6.84 (s, 1H, 3-H), 6.95 (d, 2H, 3′,5′-2H, *J* = 8.8 Hz), 7.95 (d, 2H, 2′,6′-2H, *J* = 8.8 Hz), 12.89 (s, 1H, 5-OH). ^13^C-NMR (100 MHz, DMSO-*d_6_*) δ: 24.35, 25.23, 29.11, 30.79, 41.38, 56.50, 66.15, 93.67, 98.79, 103.50, 105.22, 116.47, 121.47, 129.00, 157.63, 161.68, 161.83, 164.52, 164.58, 182.36. ESI-MS (+) for C_24_H_27_NO_5_: [M+H]^+^_(found)_ = 410.2 [M+H]^+^_(calcd)_ = 410.2.

*7-[3-(Pyrrolidin-1-yl)propoxy]-5-hydroxy-2-(4-hydroxyphenyl)-4H-chromen-4-one* (**4h**). Prepared as described for **3a**, with **2b** (0.39 g, 1 mmol) and pyrrolidine (0.71 g, 10 mmol). Yield 66%, pale yellow powder, m.p. 269–270 °C; ^1^H-NMR (400 MHz, DMSO-*d_6_*) δ: 1.97 (s, 4H), 2.15–2.22 (m, 2H), 2.98 (t, 2H, CH_2_, *J* = 6.1 Hz), 3.30–3.33 (m, 4H), 4.21 (t, 2H, CH_2_, *J* = 6.1 Hz,), 6.37 (d, 1H, 6-H, *J* = 2.1 Hz), 6.78 (d, 1H, 8-H, *J* = 2.1 Hz), 6.85 (s, 1H, 3-H), 6.96 (d, 2H, 3′,5′-2H, *J* = 8.9 Hz), 7.96 (d, 2H, 2′,6′-2H, *J* = 8.8 Hz), 10.40 (s, 1H, 4’-OH), 12.97 (s, 1H, 5-OH). ^13^C-NMR (100 MHz, DMSO-*d_6_*) δ: 23.15, 25.57, 51.63, 53.73, 66.19, 93.64, 98.82, 103.51, 105.25, 116.47, 121.47, 129.01, 157.63, 161.65, 161.83, 164.45, 164.57, 182.37. ESI-MS (+) for C_22_H_23_NO_5_: [M+H]^+^_(found)_ = 382.2 [M+H]^+^_(calcd)_ = 382.2.

*7-[3-(Morpholin-4-yl)propoxy]-5-hydroxy-2-(4-hydroxyphenyl)-4H-chromen-4-one* (**4i**). Prepared as described for **3a**, with **2b** (0.39 g, 1 mmol) and morpholine (0.87 g, 10 mmol). Yield 65%, pale yellow powder, m.p. 178–180 °C; ^1^H-NMR (400 MHz, DMSO-*d_6_*) δ: 1.87–1.94 (m, 2H, CH_2_), 2.37 (s, 4H), 2.42 (t, 2H, CH_2_, *J* = 7.0 Hz), 3.57–3.60 (m, 4H), 4.14 (t, 2H, CH_2_, *J* = 6.4 Hz), 6.35 (d, 1H, 6-H, *J* = 2.0 Hz), 6.76 (d, 1H, 8-H, *J* = 2.0 Hz), 6.83 (s, 1H, 3-H), 6.94 (d, 2H, 3′,5′-2H, *J* = 8.8 Hz), 7.96 (d, 2H, 2′,6′-2H, *J* = 8.8 Hz), 12.94 (s, 1H, 5-OH). ^13^C-NMR (100 MHz, DMSO-*d_6_*) δ: 26.10, 53.83, 55.07, 66.69, 67.22, 93.48, 98.78, 103.44, 105.09, 116.45, 121.48, 129.01, 157.70, 161.64, 161.86, 164.52, 164.95, 182.36. ESI-MS (−) for C_22_H_23_NO_6_: [M-H]^–^_(found)_ = 396.1 [M-H]^–^_(calcd)_ = 396.2.

*7-[3-(Piperazin-1-yl)propoxy]-5-hydroxy-2-(4-hydroxyphenyl)-4H-chromen-4-one* (**4j**). Prepared as described for **3a**, with **2b** (0.39 g, 1 mmol) and piperazine (0.86 g, 10 mmol). Yield 70%, pale yellow powder, m.p. 164–166 °C; ^1^H-NMR (400 MHz, DMSO-*d_6_*) δ: 1.89–1.92 (m, 2H, CH_2_), 2.50–2.51 (m, 2H), 2.61 (br s, 4H), 3.08–3.10 (m, 4H), 4.14 (t, 2H, CH_2_, *J* = 6.2 Hz), 6.36 (d, 1H, 6-H, *J* = 2.1 Hz), 6.76 (d, 1H, 8-H, *J* = 2.1 Hz,), 6.84 (s, 1H, 3-H), 6.95 (d, 2H, 3′,5′-2H, *J* = 8.8 Hz), 7.96 (d, 2H, 2′,6′-2H, *J* = 8.8 Hz), 12.94 (s, 1H, 5-OH). ^13^C-NMR (100 MHz, DMSO-*d_6_*) δ: 26.16, 43.48, 49.76, 54.22, 67.05, 93.55, 98.77, 103.47, 105.10, 116.46, 121.50, 129.01, 157.69, 161.64, 161.80, 164.54, 164.91, 182.35. ESI-MS (+) for C_22_H_24_N_2_O_5_: [M+H]^+^_(found)_ = 397.2 [M+H]^+^_(calcd)_ = 397.2.

### 3.3. Antibacterial Assay

Screening of the synthesized compounds for antibacterial activity was performed using the disk diffusion method, according to the National Committee for Clinical Laboratory Standards (NCCLS) [23,24], which is normally used as a preliminary test. Prior to the experiment, sterile paper discs (6.0 mm in diameter), impregnated with compound dissolved in DMSO at a concentration of 1000 μg/mL, were prepared. Sterile LB agar medium (10 mL) was then mixed with 10 μL of bacterial suspension at 40 °C and poured onto an agar plate. The impregnated paper discs were placed on the surface of the media inoculated with the microorganism. Ampicillin and tetracycline were used as positive controls, and DMSO was used as negative control. After 18 h of incubation at 37 °C, the diameter of the zone of growth inhibition around the disc was measured. The average of three parallel experiments was recorded.

MIC was defined as the lowest concentration of compound in which bacterial growth after incubation was completely inhibited. MIC values for the synthesized compounds were determined by NCCLS method using LB medium in 96-well tissue culture plates [25]. Antibacterial activity was evaluated against two Gram-positive bacteria, namely, *S. aureus* and *B. subtilis*, and two Gram-negative bacteria, namely, *E. coli* and *P. aeruginosa*. Tetracycline and ampicillin were also used as positive controls. They were dissolved in DMSO at a concentration of 2.5 mg/mL, and 20 μL of this solution was pipetted to the first well of each line in 96-well tissue culture plate with 180 μL LB medium. The solution was then serially diluted to afford two-fold serial dilutions of the test compounds and positive controls in the subsequent wells which contain 100 μL LB medium. Afterwards, 100 μL bacterial suspension (10^6^ CFU/mL) was added to each well and kept for incubation. One well containing bacterial cells and DMSO without any test compound was used as growth control, and another well containing only growth medium was used as blank control. The maximum concentration of tested compounds was 125 μg/mL. MIC values were recorded spectrophotometrically in 630 nm after 24 h incubation at 37 °C.

### 3.4. Antiproliferative Assay

The antiproliferative activities of all the synthesized apigenin derivatives were evaluated against A549, HeLa, HepG2, and MCF-7 cells by MTT staining according to the procedures reported previously [26,27], with slight modification.

Briefly, target cancer cells were grown to log phase in a suitable medium supplemented with 10% FBS, 100 U/mL penicillin, and 100 μg/mL streptomycin at 37 °C and 5% CO_2_ with 95% humidity. The cells were harvested through trypsinization with 0.25% trypsin in Ca^2+^- and Mg^2+^-free Hanks’ balanced salt solution at 37 °C. Trypsinization was stopped by adding fresh supplemented medium. The cell suspension was centrifuged at 1000 rpm for 5 min, and then the cells were resuspended in supplemented medium (1.0 × 10^6^ cells/well in 6-well plates) for further use. Afterward, the cells were seeded into 96-well microtiter plates (4.0 × 10^3^ cells/well) with fresh medium (150 μL). Subsequent incubation was permitted for 24 h before the antiproliferative assessments. Tested compounds of 150 μL (final concentrations of 62.5, 125, 250, 500, 1,000, or 2,000 μg/mL in the culture medium) were added to each well. After 24 h incubation, MTT (20 μL, 5 mg/mL) was added to each well. After 4 h, DMSO (150 μL) was added to terminate the reaction. The survival rate of the cancer cells was evaluated by measuring the optical density (A) on a microplate reader at 490 nm. All *in vitro* results were expressed as the cancer cell proliferation inhibition ratio according to the following formula:

[(*A_control_* − *A_test_*)/*A_control_*] × 100%

where *A_control_* and *A_test_* are the optical densities of the control and the test groups, respectively. All tests were performed in triplicate.

## 4. Conclusions

In summary, two series of apigenin derivatives, which contains 2- or 3-carbon spacers, were synthesized, and their antibacterial and antiproliferative activities were assessed. Most of the synthesized derivatives showed potential antibacterial and antiproliferative activities. Among these derivatives, **4a**–**4j**, which contain a C_3_ spacer between apigenin and the different amines, displayed greater activity than **3a**–**3j**. Compound **4i** was found to be the most active derivative, with MIC values of 1.95, 3.91, 3.91, and 3.91 μg/mL against *S. aureus*, *B. subtilis*, *E. coli*, and *P. aeruginosa*, respectively. Compound **4j** showed notable activity against A549, HeLa, HepG2, and MCF-7 cancer cell lines, with the lowest IC_50_ values among all the synthesized compounds. These findings have encouraged us to continue the development and testing of apigenin derivatives and conduct further studies to investigate SARs and their mechanisms of action.

## Supplementary Materials

^1^H-NMR and ^13^C-NMR spectra of the compounds **1**, **2a**, **2b**, **3a**–**3j**, and **4a**–**4j** are available in the supplementary materials: http://www.mdpi.com/1420-3049/18/9/11496/s1.
