# Supplementary material for: Synthesis and Biological Evaluation of Apigenin Derivatives as Antibacterial and Antiproliferative Agents
Source: Molecules. 2013 Sep 17;18(9):11496–511. doi: 10.3390/molecules180911496 (PMC6269912; doi:10.3390/molecules180911496)

Supplementary Materials

Figure S1. ^{1}H-NMR spectrum (500 MHz, DMSO-d_6) of 7-(2-bromoethoxy)-5-hydroxy-2-(4-hydroxyphenyl)-4H-chromen-4-one **2a**.

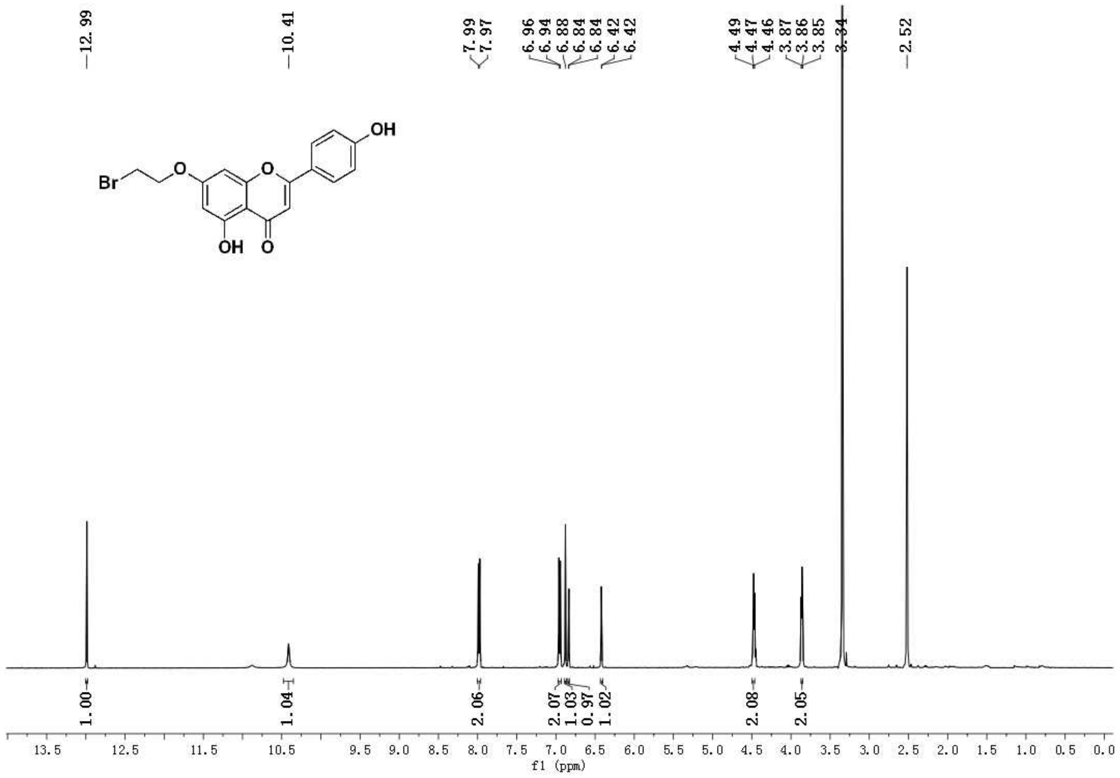

Figure S2. ^{13}C-NMR spectrum (125 MHz, DMSO-d_6) of 7-(2-bromoethoxy)-5-hydroxy-2-(4-hydroxyphenyl)-4H-chromen-4-one **2a**.

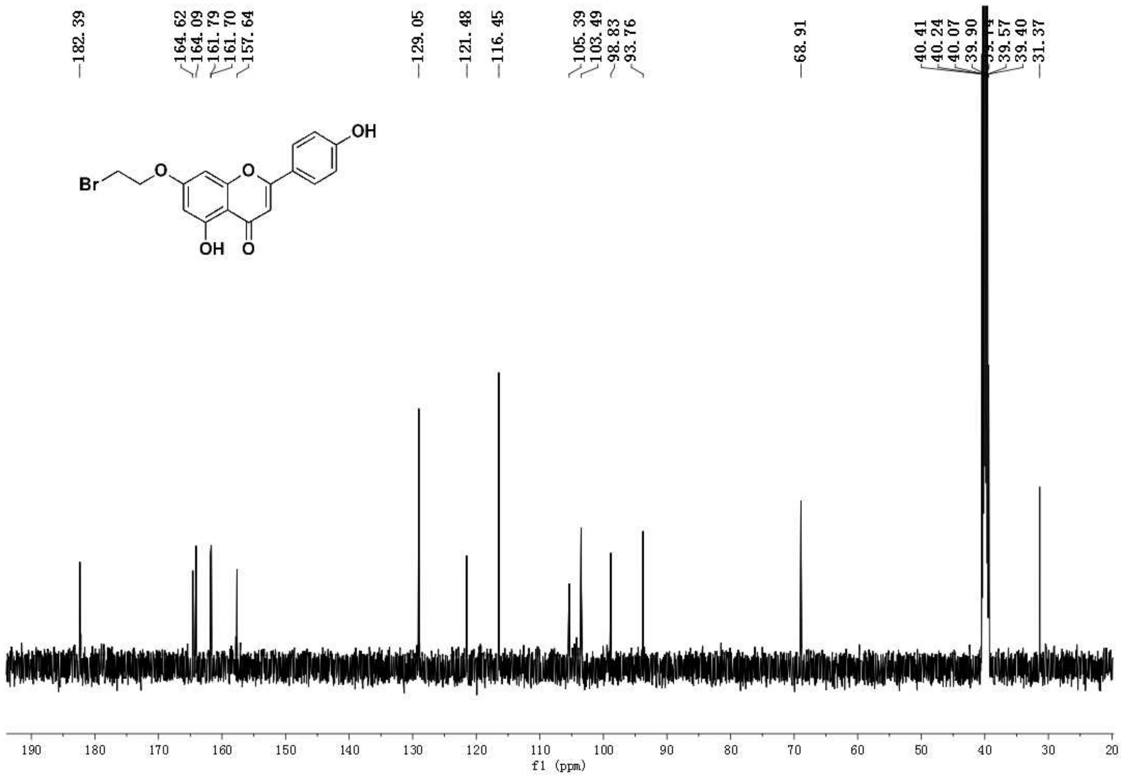

Figure S3. ^{1}H-NMR spectrum (500 MHz, DMSO-d_6) of 7-(3-bromopropoxy)-5-hydroxy-2-(4-hydroxyphenyl)-4H-chromen-4-one **2b**.

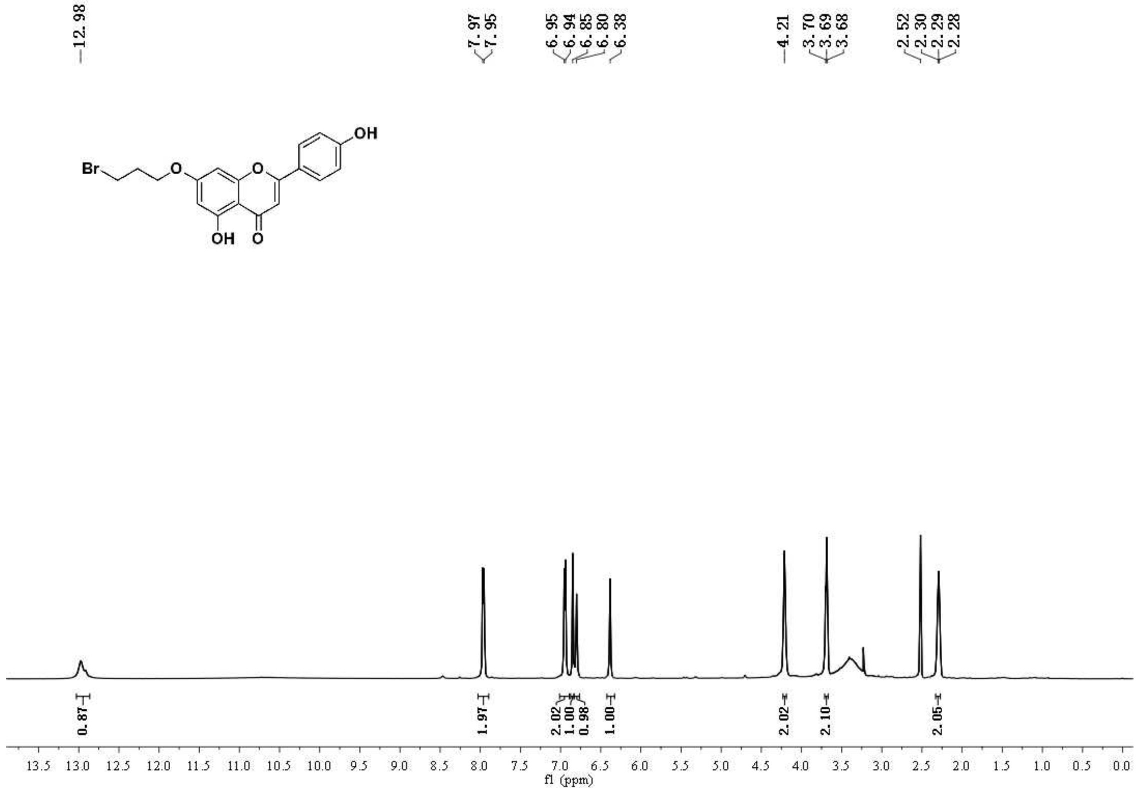

Figure S4. ^{13}C-NMR spectrum (125 MHz, DMSO-d_6) of 7-(3-bromopropoxy)-5-hydroxy-2-(4-hydroxyphenyl)-4H-chromen-4-one **2b**.

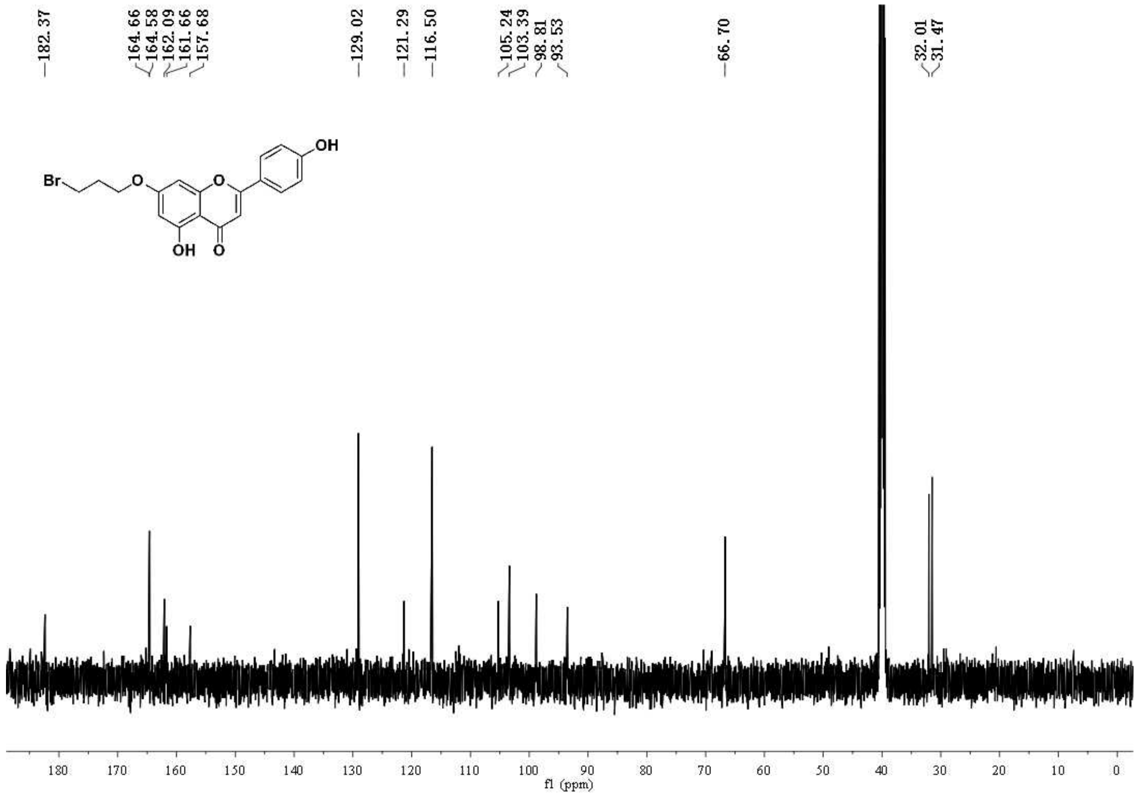

Figure S5. ^{1}H-NMR spectrum (500 MHz, DMSO-d_6) of 7-[2-(diethylamino)ethoxy]-5-hydroxy-2-(4-hydroxyphenyl)-4H-chromen-4-one **3a**.

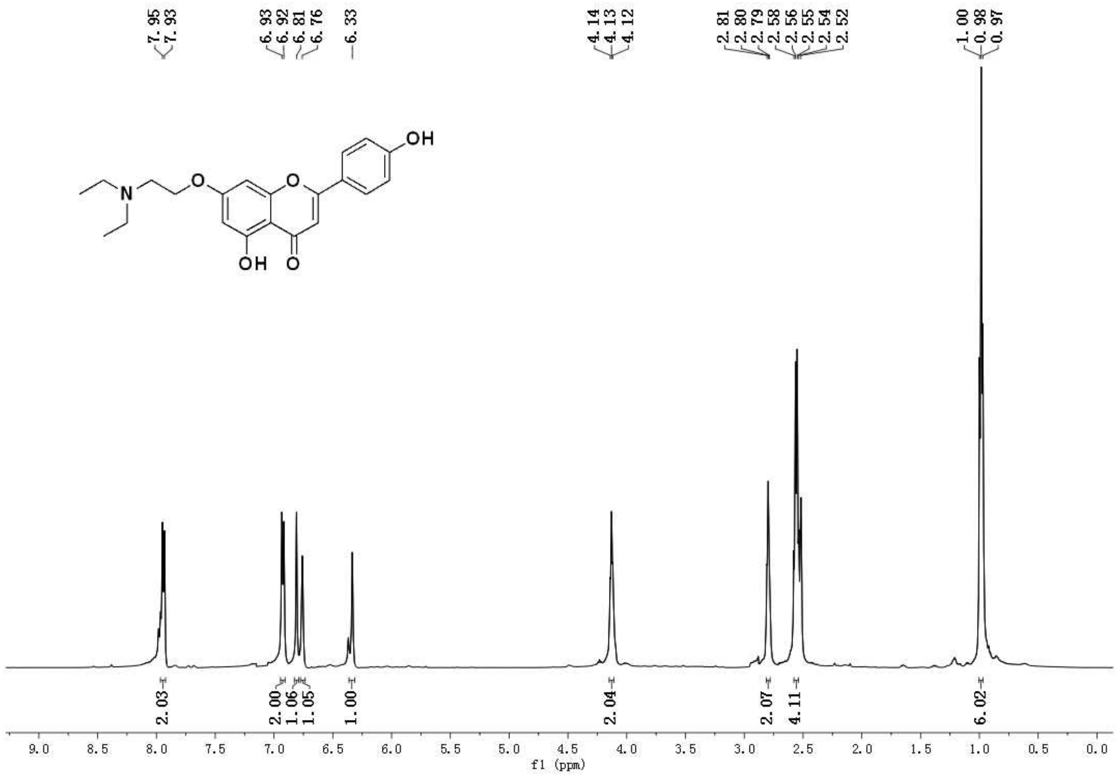

Figure S6. ^{13}C-NMR spectrum (125 MHz, DMSO-d_6) of 7-[2-(diethylamino)ethoxy]-5-hydroxy-2-(4-hydroxyphenyl)-4H-chromen-4-one **3a**.

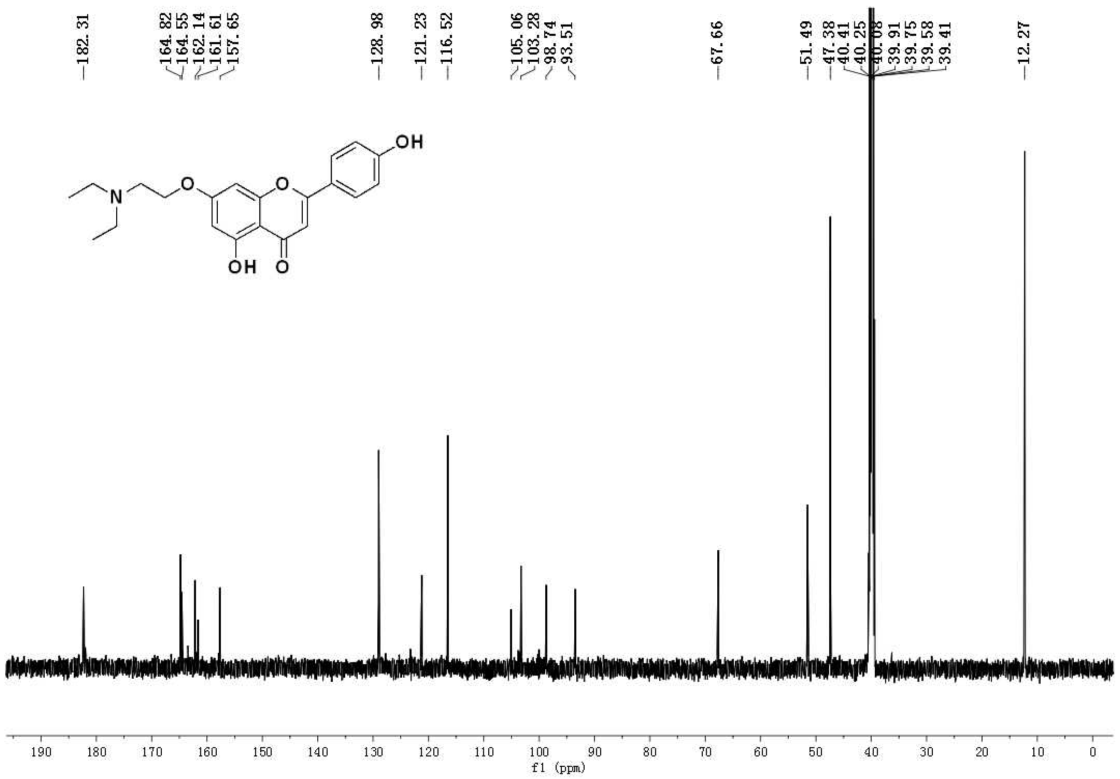

Figure S7. ^{1}H-NMR spectrum (500 MHz, DMSO-d_6) of 7-[2-(ethylamino)ethoxy]-5-hydroxy-2-(4-hydroxyphenyl)-4H-chromen-4-one **3b**.

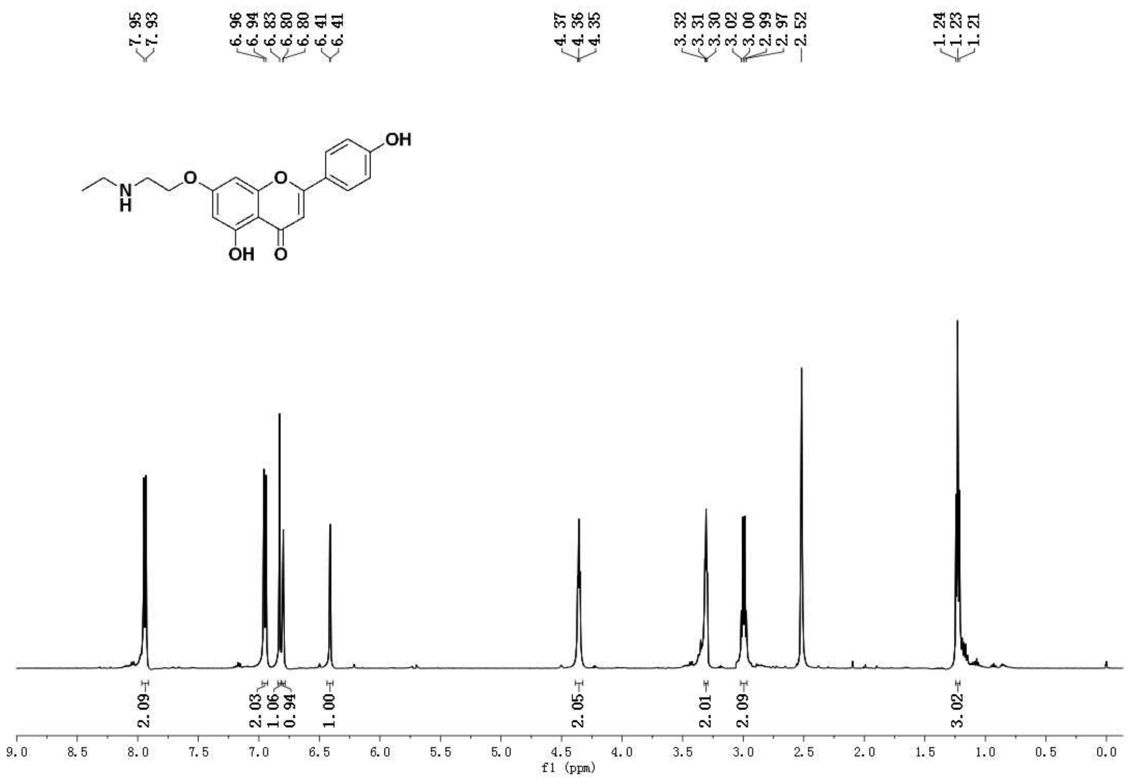

Figure S8. ^{13}C-NMR spectrum (125 MHz, DMSO-d_6) of 7-[2-(ethylamino)ethoxy]-5-hydroxy-2-(4-hydroxyphenyl)-4H-chromen-4-one **3b**.

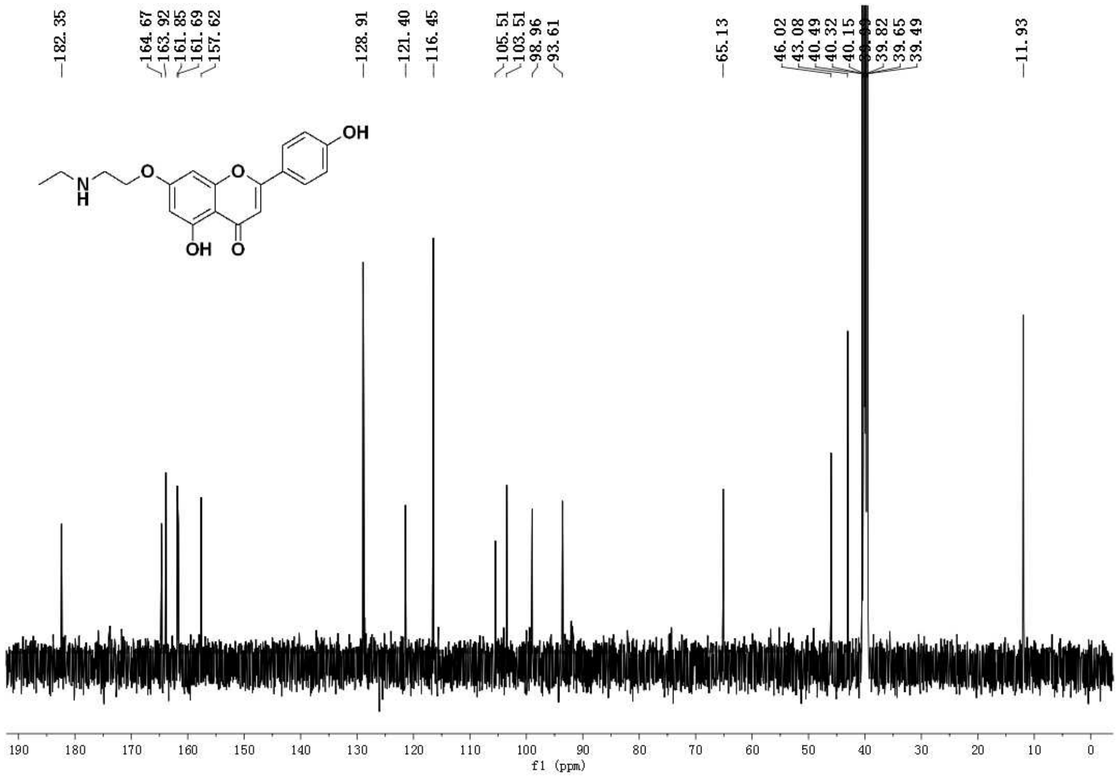

Figure S9. ^{1}H-NMR spectrum (500 MHz, DMSO-d_6) of 7-[2-(propylamino)ethoxy]-5-hydroxy-2-(4-hydroxyphenyl)-4H-chromen-4-one **3c**.

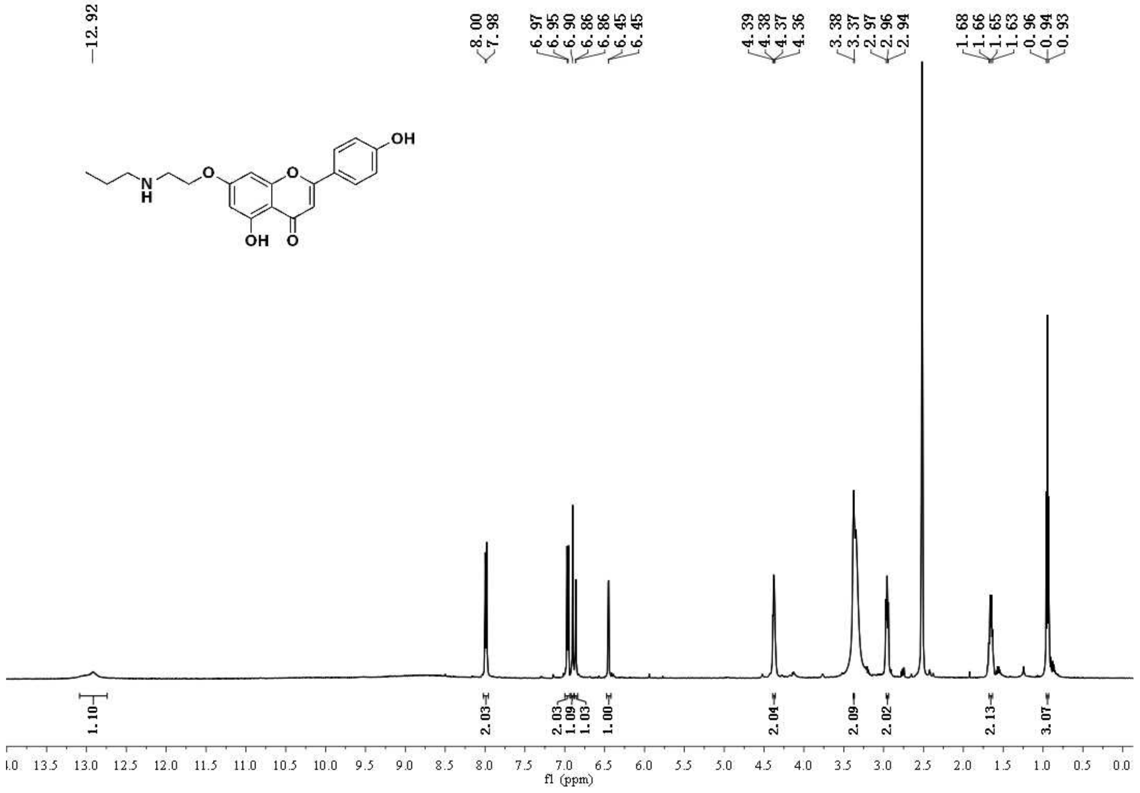

Figure S10. ^{13}C-NMR spectrum (125 MHz, DMSO-d_6) of 7-[2-(propylamino)ethoxy]-5-hydroxy-2-(4-hydroxyphenyl)-4H-chromen-4-one **3c**.

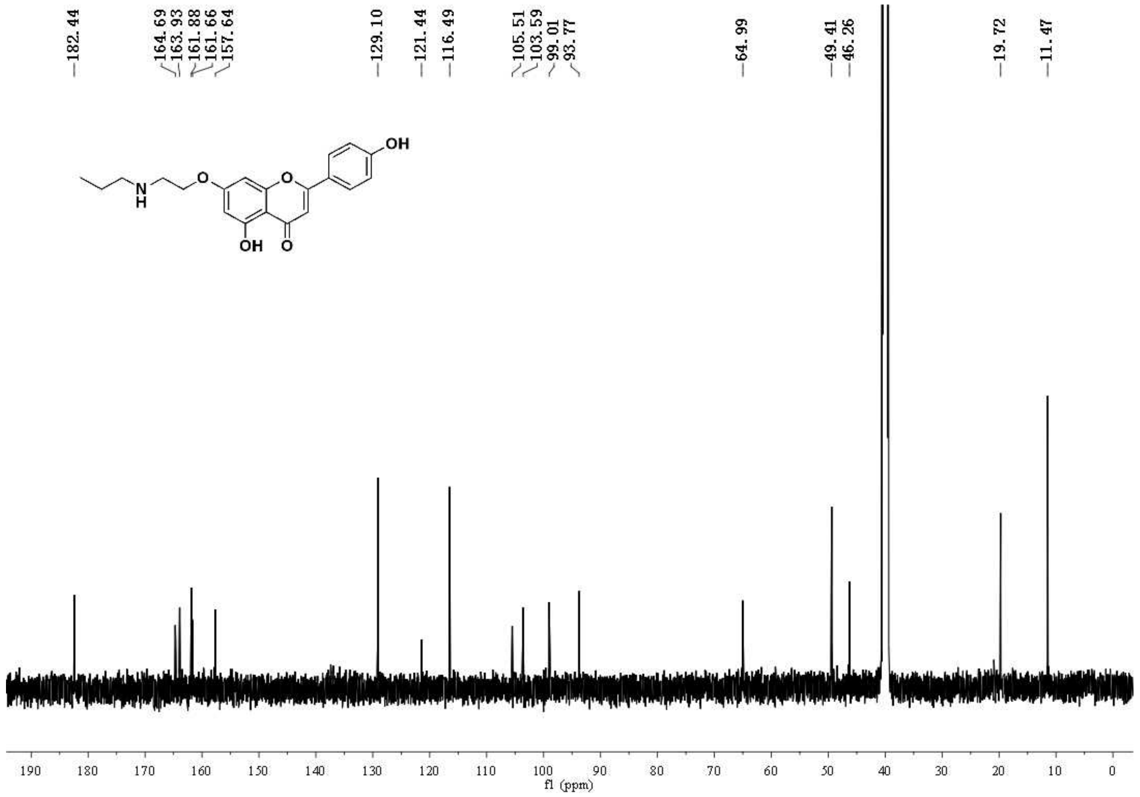

Figure S11. ^{1}H-NMR spectrum (500 MHz, DMSO-d_6) of 7-[2-(*n*-butylamino)ethoxy]-5-hydroxy-2-(4-hydroxyphenyl)-4H-chromen-4-one **3d**.

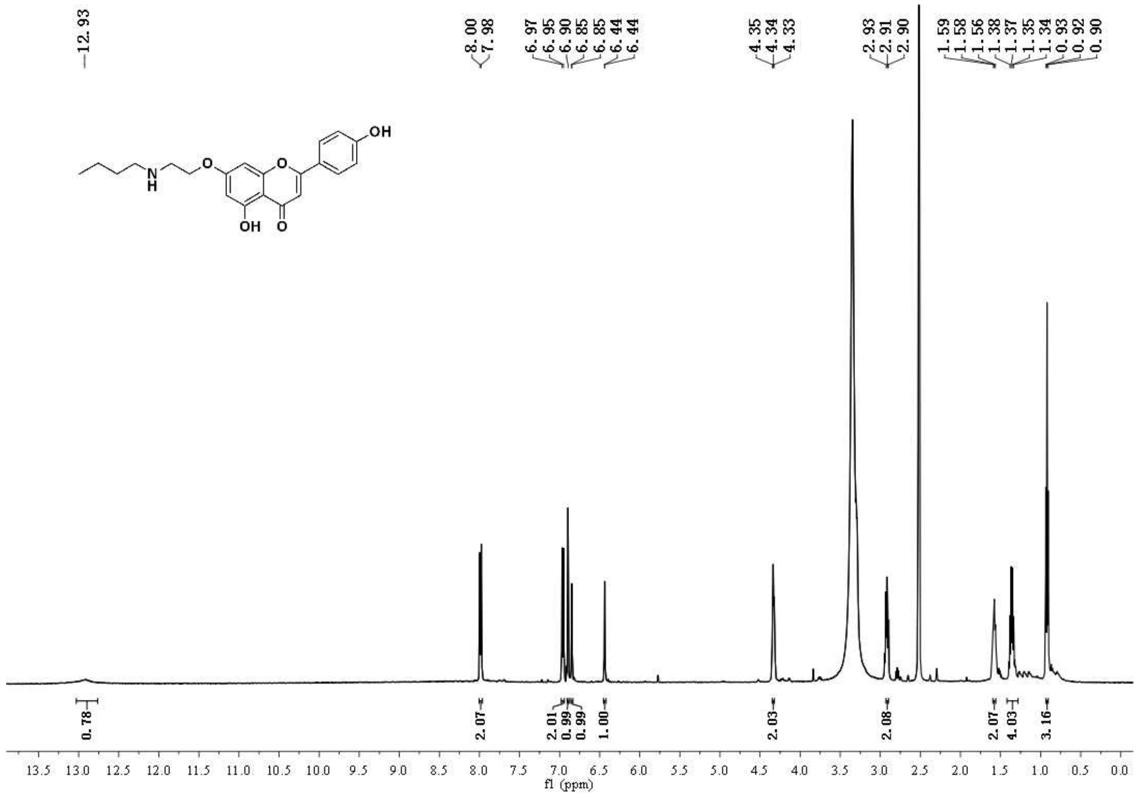

Figure S12. ^{13}C-NMR spectrum (125 MHz, DMSO-d_6) of 7-[2-(*n*-butylamino)ethoxy]-5-hydroxy-2-(4-hydroxyphenyl)-4H-chromen-4-one **3d**.

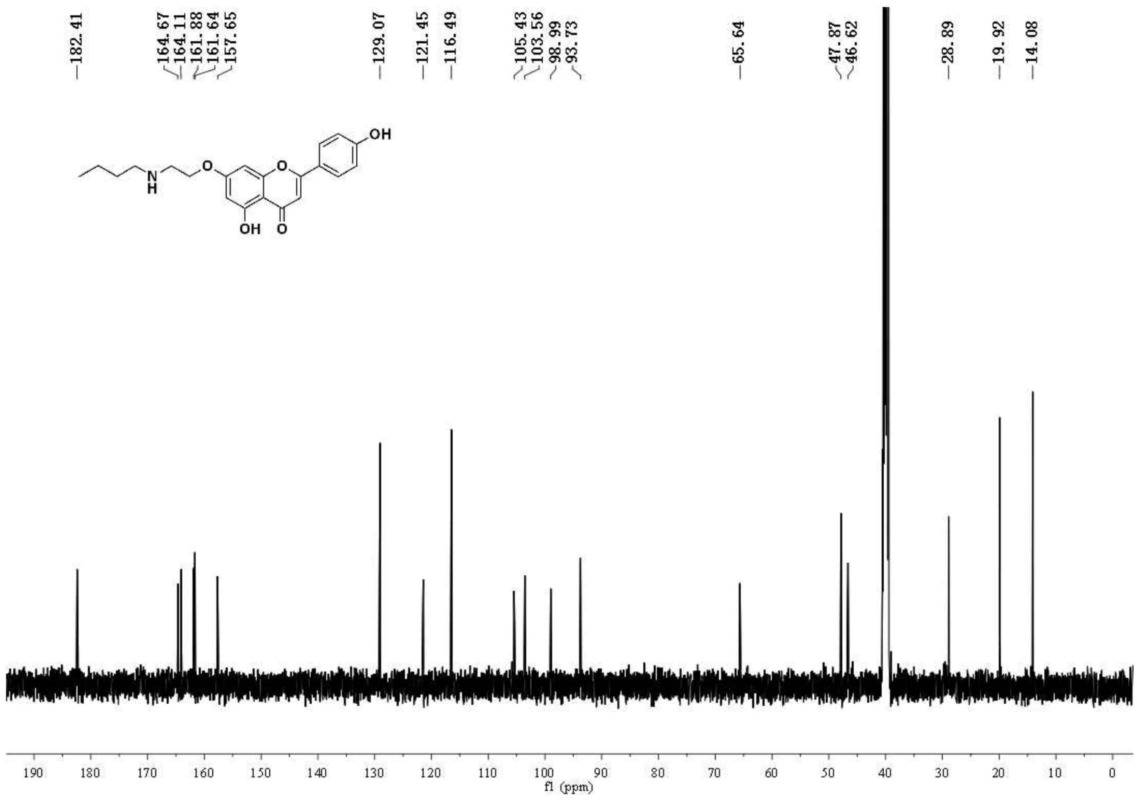

Figure S13. ^{1}H-NMR spectrum (500 MHz, DMSO-d_6) of 7-[2-(*tret*-butylamino)ethoxy]-5-hydroxy-2-(4-hydroxyphenyl)-4H-chromen-4-one **3e**.

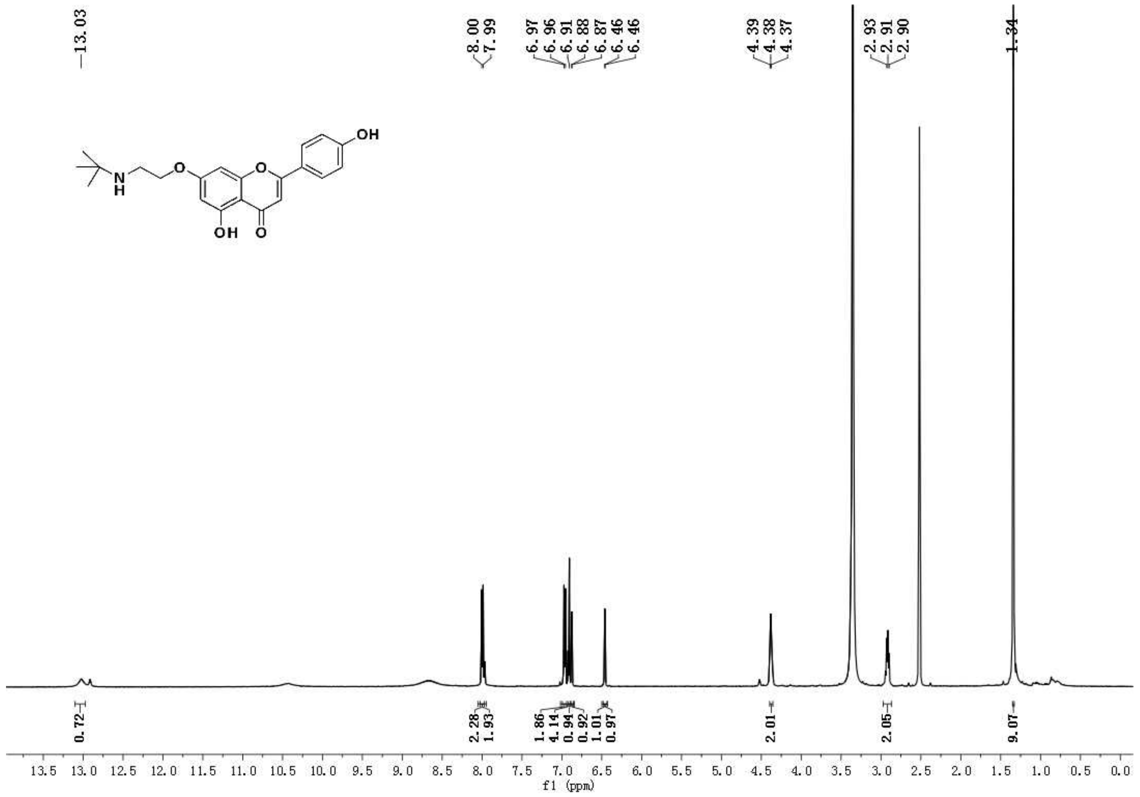

Figure S14. ^{13}C-NMR spectrum (125 MHz, DMSO-d_6) of 7-[2-(*tret*-butylamino)ethoxy]-5-hydroxy-2-(4-hydroxyphenyl)-4H-chromen-4-one **3e**.

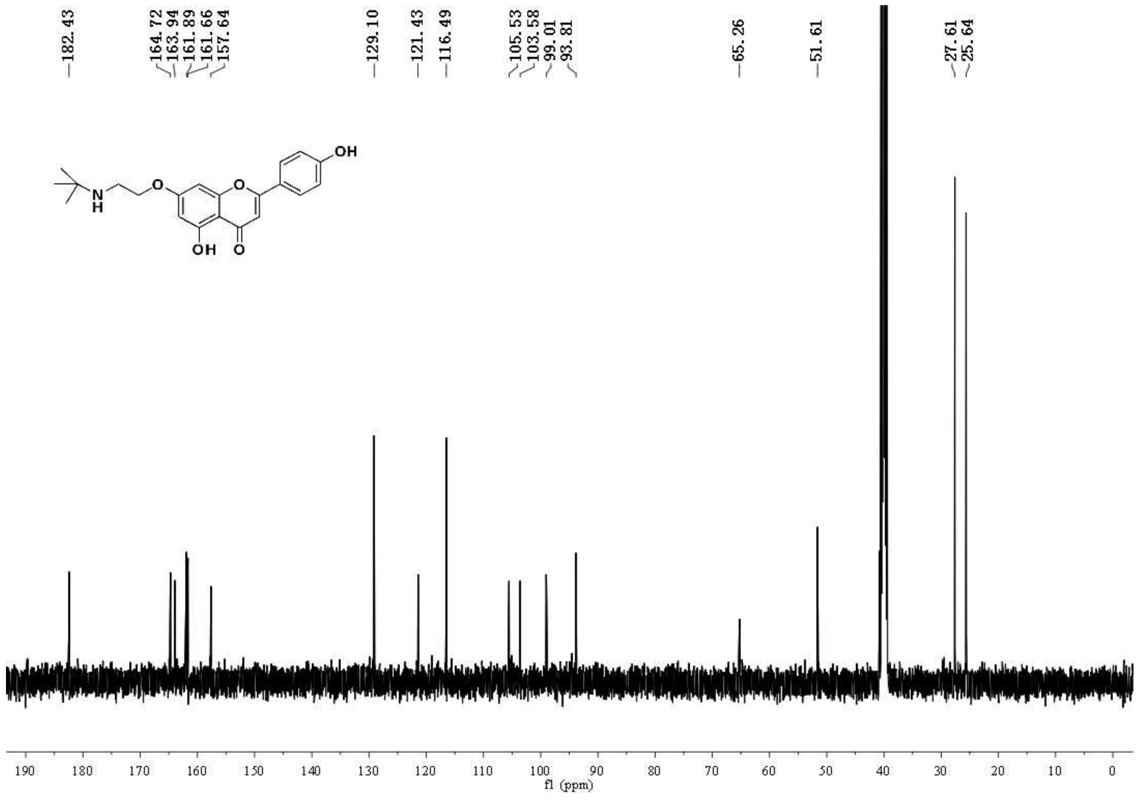

Figure S15. ^{1}H-NMR spectrum (500 MHz, DMSO-d_6) of 7-[2-(2'-hydroxyethylamino)ethoxy]-5- hydroxy-2-(4-hydroxyphenyl)-4H-chromen-4-one **3f**.

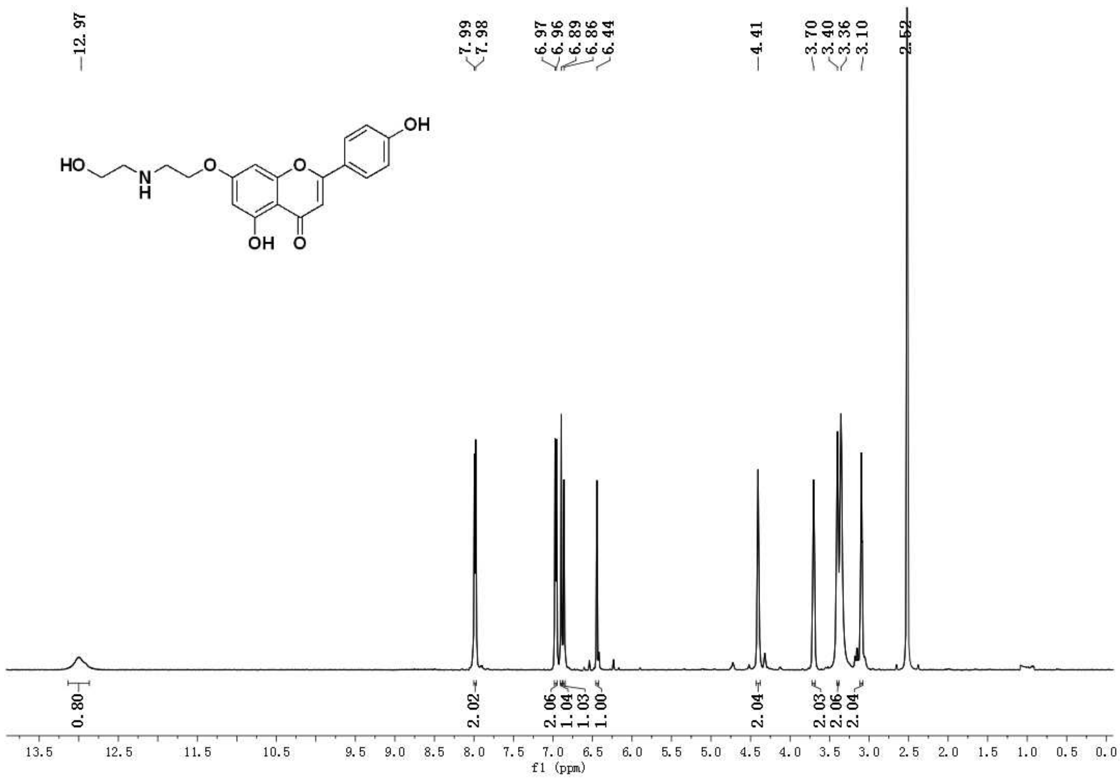

Figure S16. ^{13}C-NMR spectrum (125 MHz, DMSO-d_6) of 7-[2-(2'-hydroxyethylamino)ethoxy]-5- hydroxy-2-(4-hydroxyphenyl)-4H-chromen-4-one **3f**.

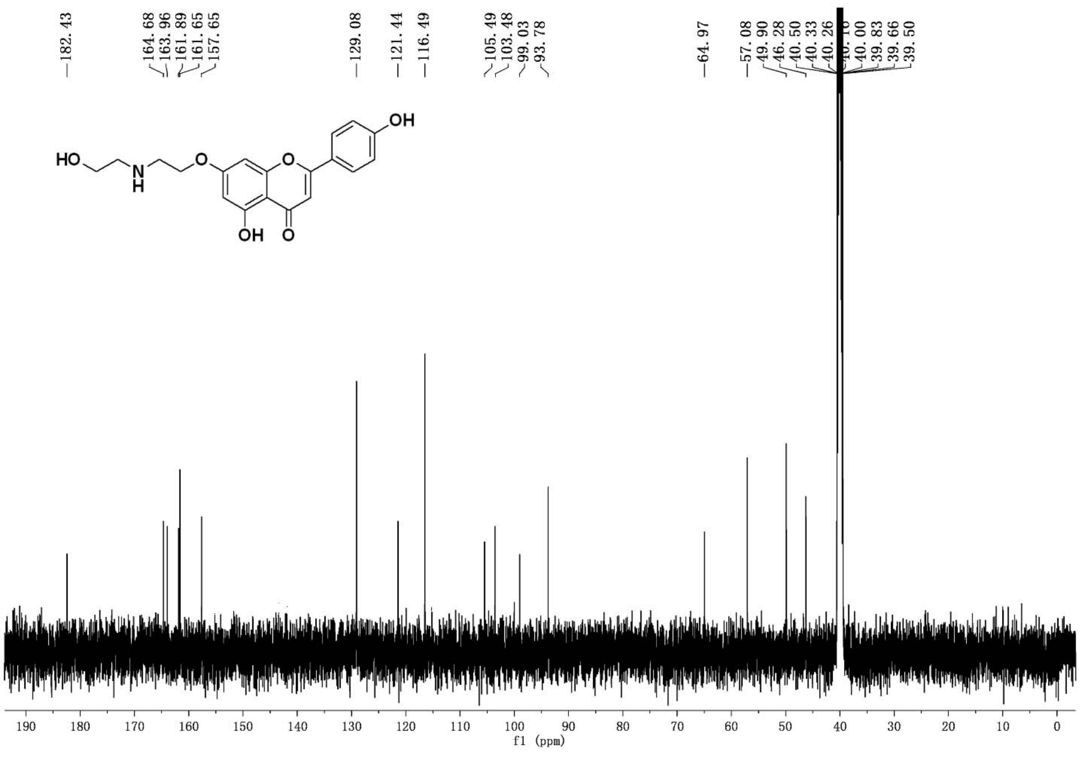

Figure S17. ^{1}H-NMR spectrum (500 MHz, DMSO-d_6) of 7-[2-(cyclohexylamino)ethoxy]-5-hydroxy-2- (4-hydroxyphenyl)-4H-chromen-4-one **3g**.

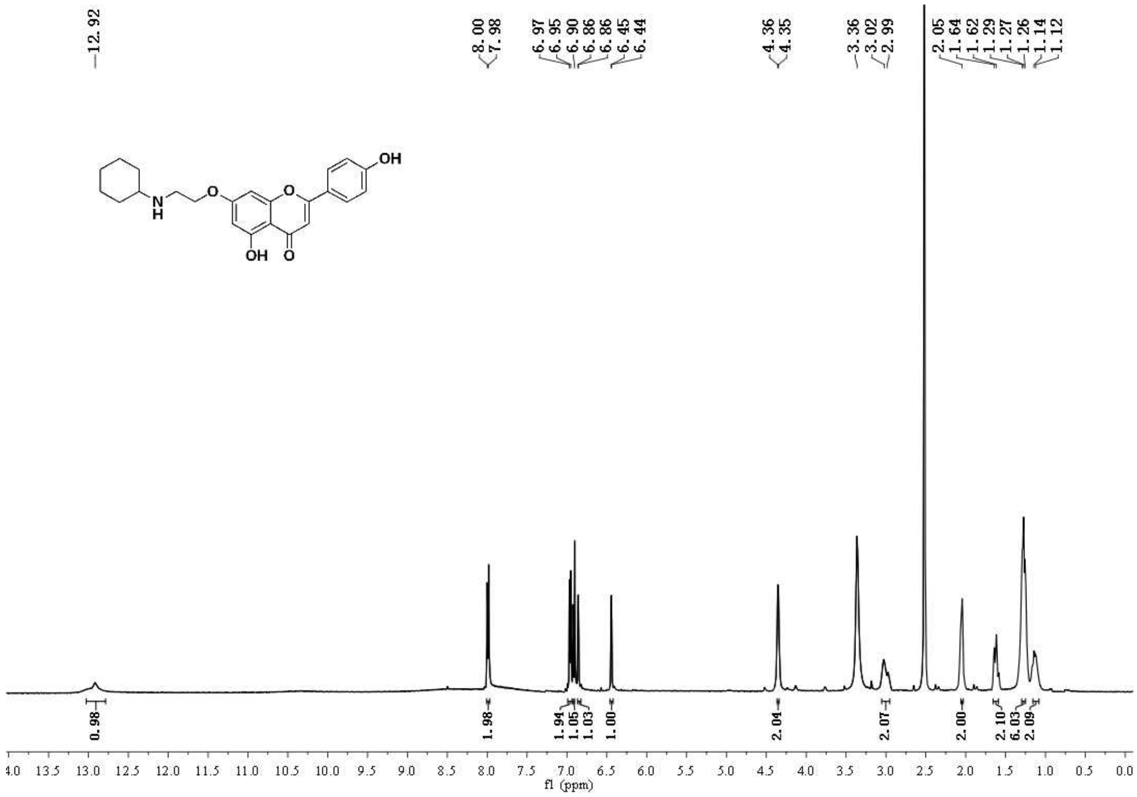

Figure S18. ^{13}C-NMR spectrum (125 MHz, DMSO-d_6) of 7-[2-(cyclohexylamino)ethoxy]-5-hydroxy-2- (4-hydroxyphenyl)-4H-chromen-4-one **3g**.

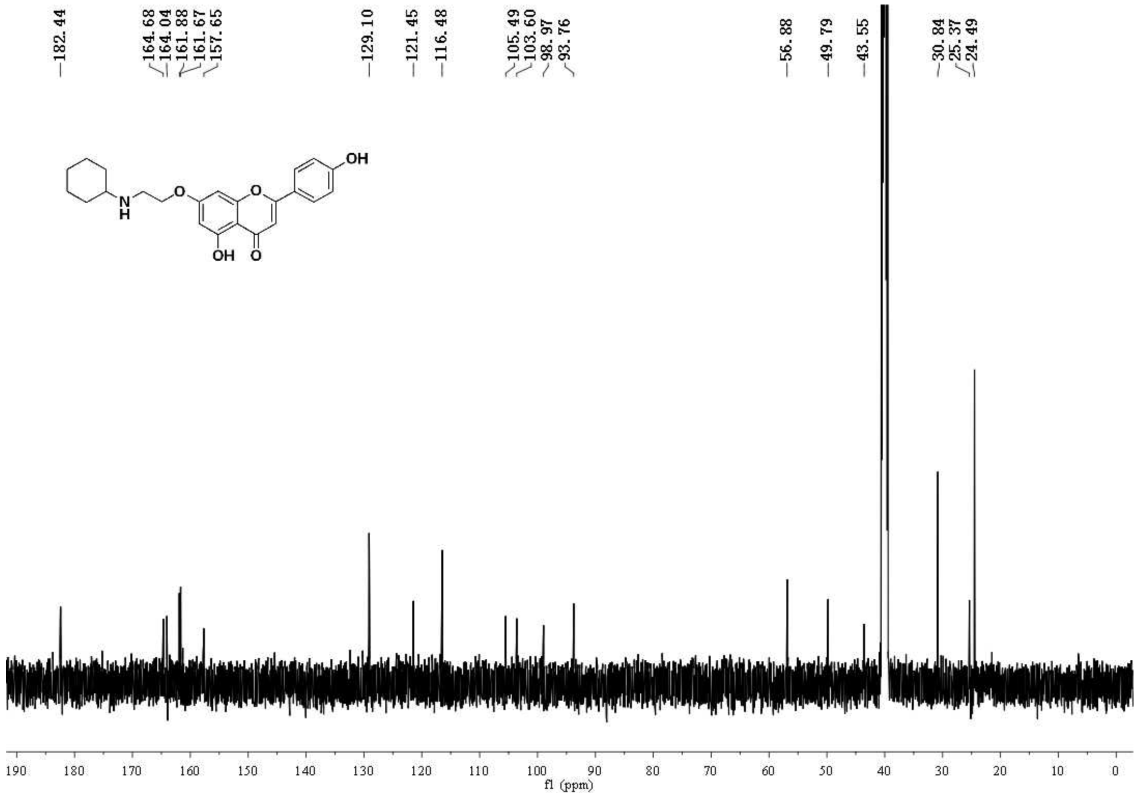

Figure S19. ^{1}H-NMR spectrum (500 MHz, DMSO-d_6) of 7-[2-(pyrrolidin-1-yl)ethoxy]-5-hydroxy-2-(4-hydroxyphenyl)-4H-chromen-4-one **3h**.

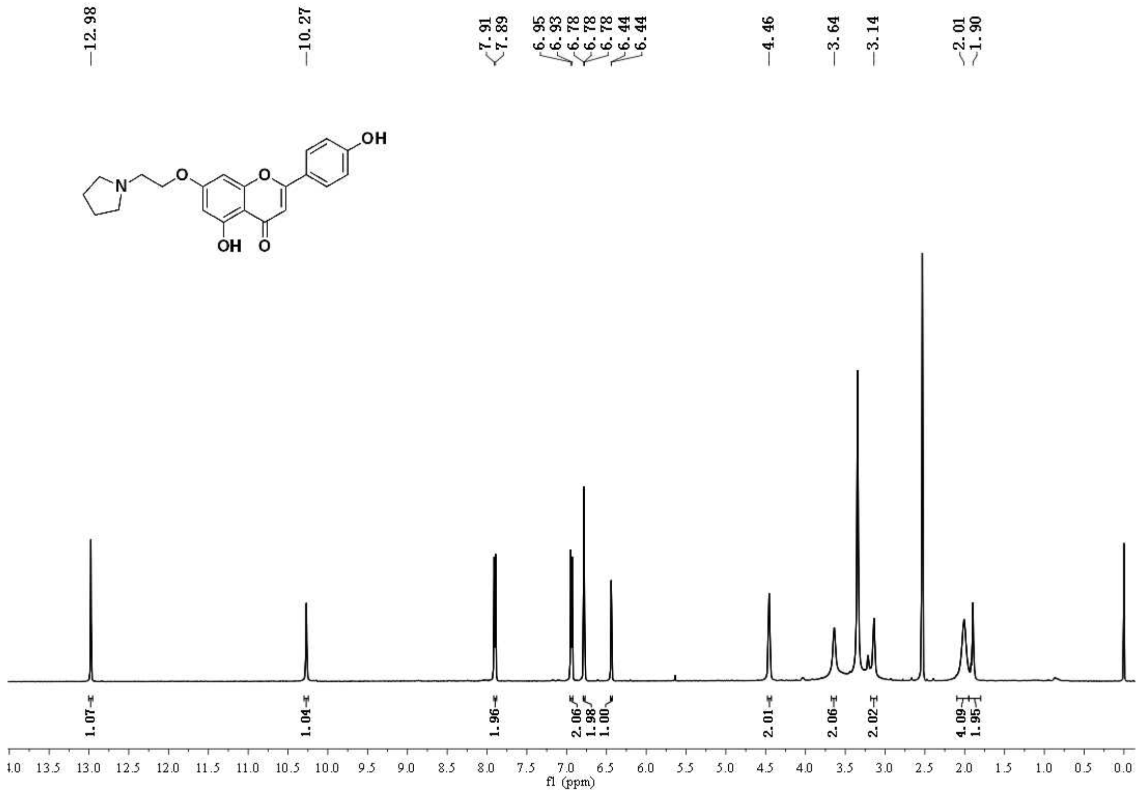

Figure S20. ^{13}C-NMR spectrum (125 MHz, DMSO-d_6) of 7-[2-(pyrrolidin-1-yl)ethoxy]-5-hydroxy-2-(4-hydroxyphenyl)-4H-chromen-4-one **3h**.

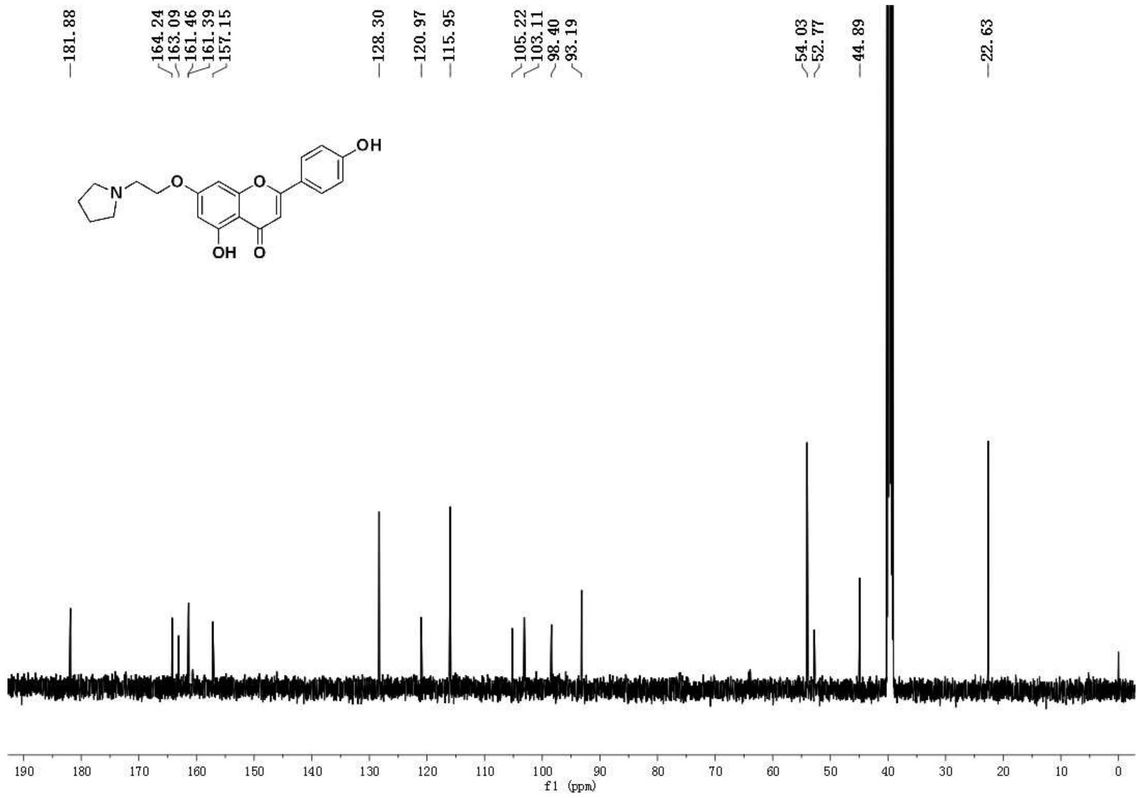

Figure S21. ^{1}H-NMR spectrum (500 MHz, DMSO-d_6) of 7-[2-(morpholin-4-yl)ethoxy]-5-hydroxy-2-(4-hydroxyphenyl)-4H-chromen-4-one **3i**.

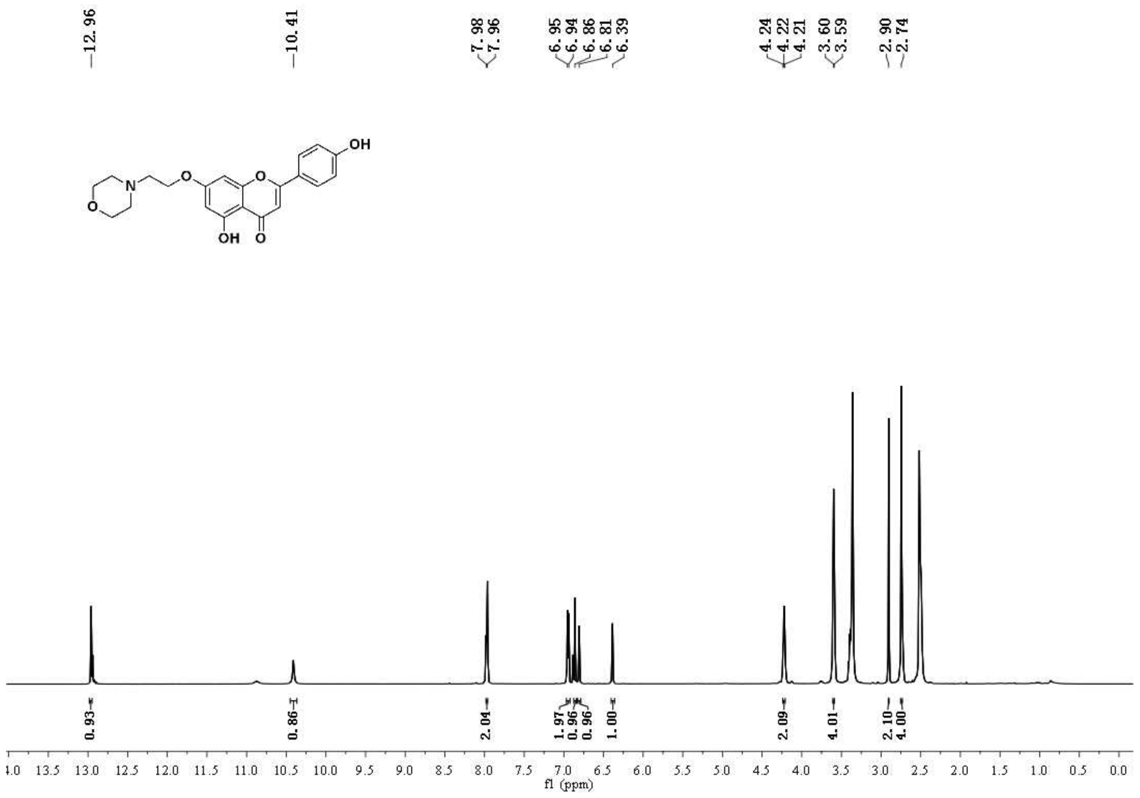

Figure S22. ^{13}C-NMR spectrum (125 MHz, DMSO-d_6) of 7-[2-(morpholin-4-yl)ethoxy]-5-hydroxy-2-(4-hydroxyphenyl)-4H-chromen-4-one **3i**.

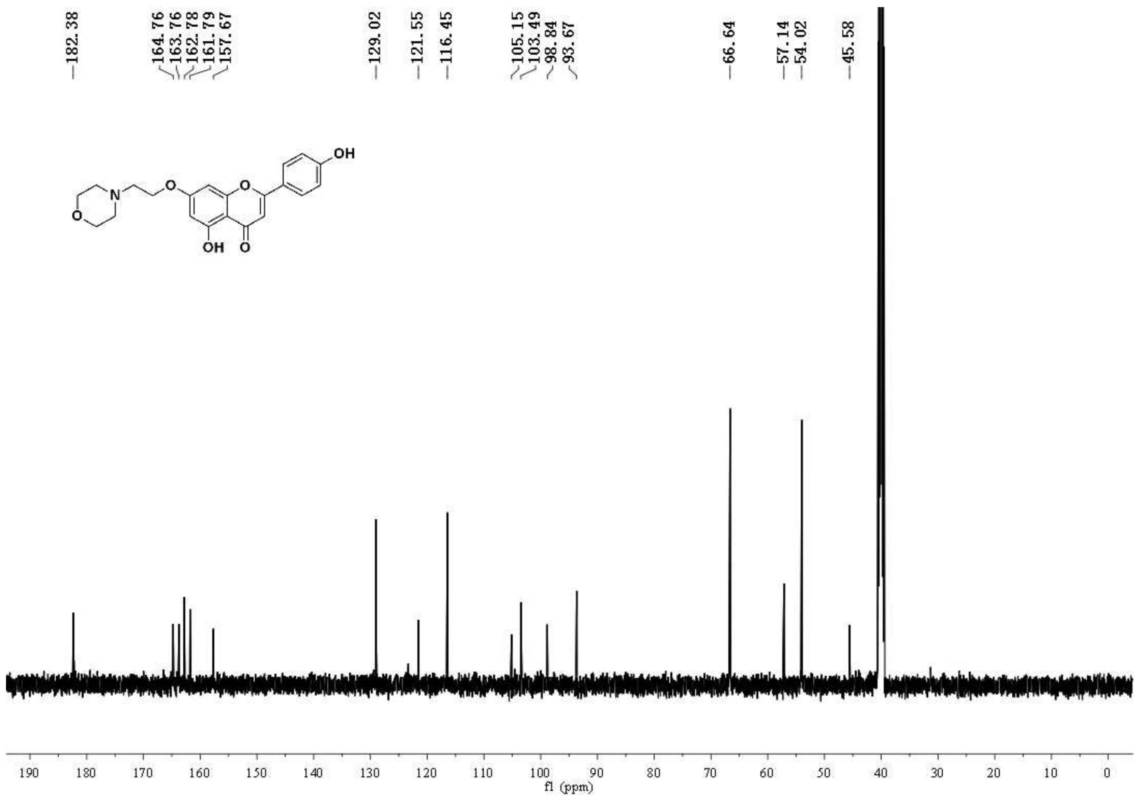

Figure S23. ^{1}H-NMR spectrum (500 MHz, DMSO-d_6) of 7-[2-(piperazin-1-yl)ethoxy]-5-hydroxy-2-(4-hydroxyphenyl)-4H-chromen-4-one **3j**.

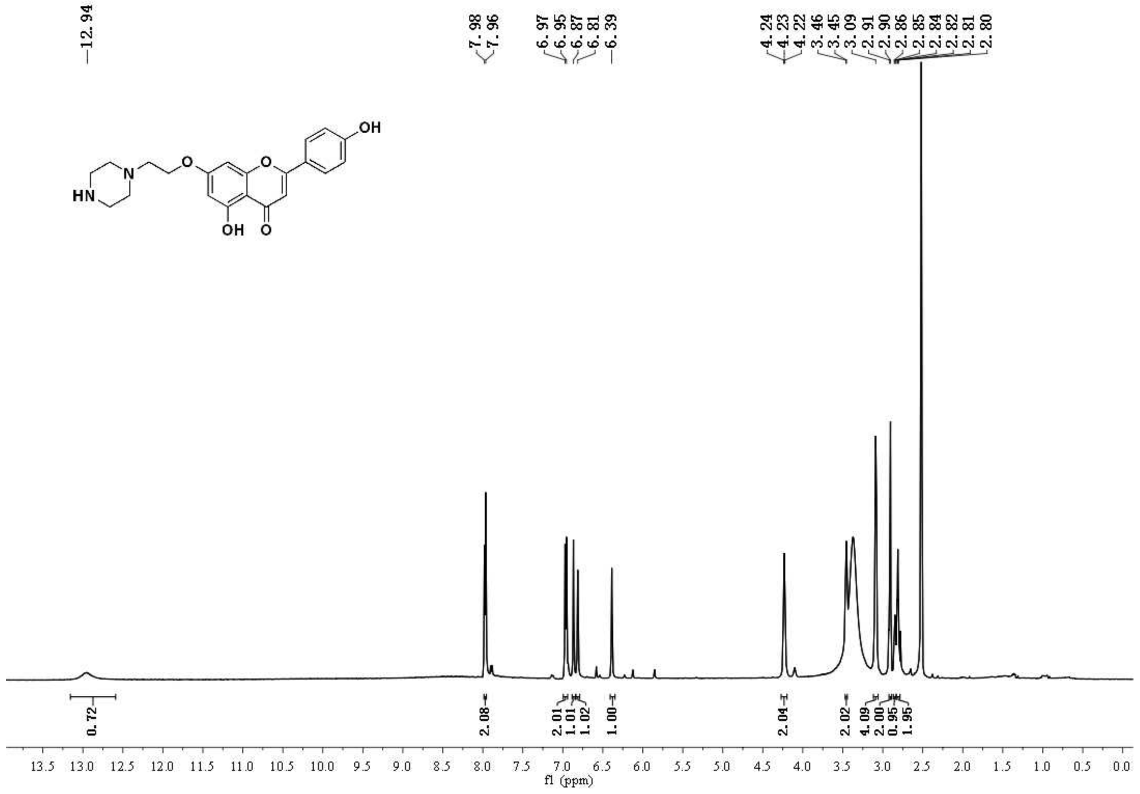

Figure S24. ^{13}C-NMR spectrum (125 MHz, DMSO-d_6) of 7-[2-(piperazin-1-yl)ethoxy]-5-hydroxy-2-(4-hydroxyphenyl)-4H-chromen-4-one **3j**.

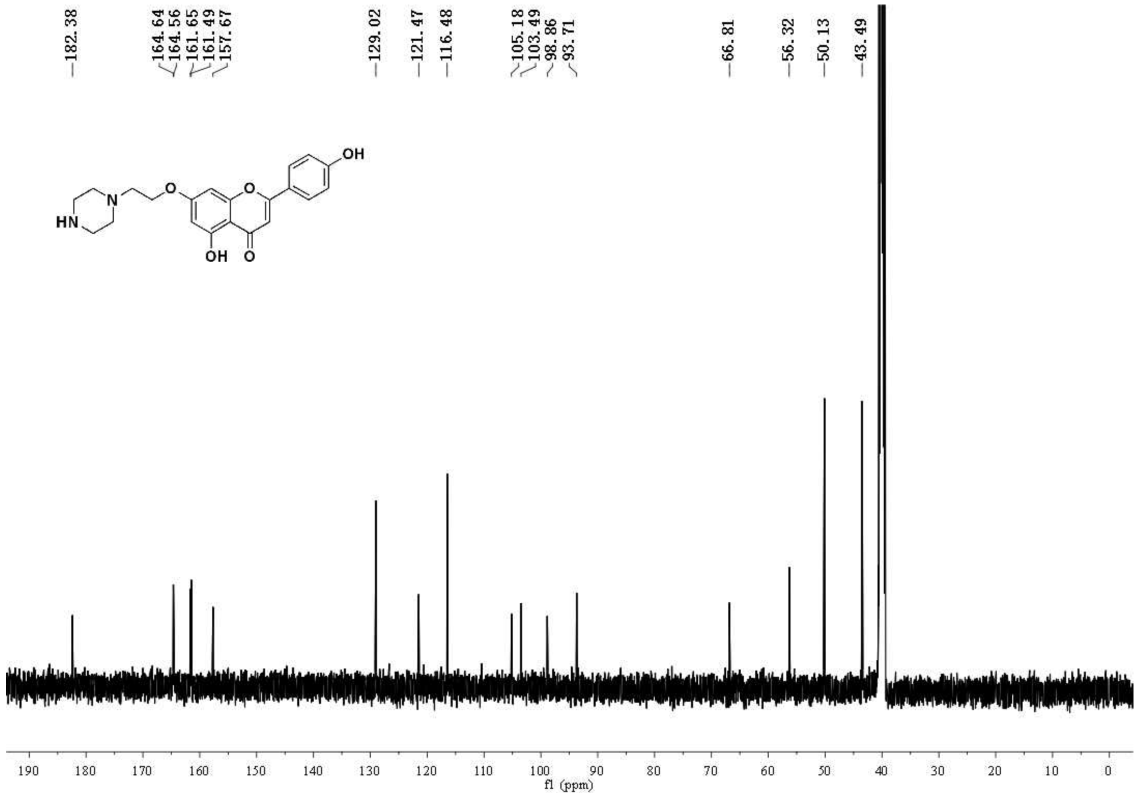

Figure S25. ^{1}H-NMR spectrum (500 MHz, DMSO-d_6) of 7-[3-(ethylamino)propoxy]-5-hydroxy-2-(4-hydroxyphenyl)-4H-chromen-4-one **4a**.

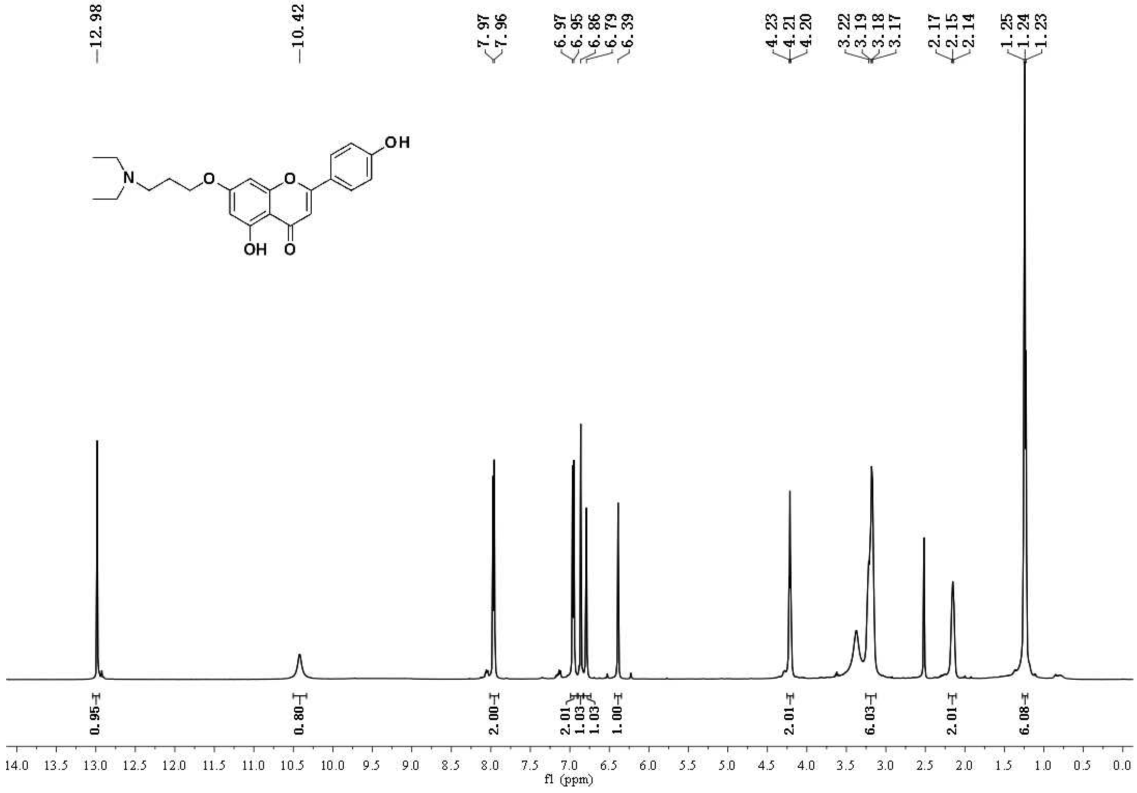

Figure S26. ^{13}C-NMR spectrum (125 MHz, DMSO-d_6) of 7-[3-(diethylamino)propoxy]-5-hydroxy-2-(4-hydroxyphenyl)-4H-chromen-4-one **4a**.

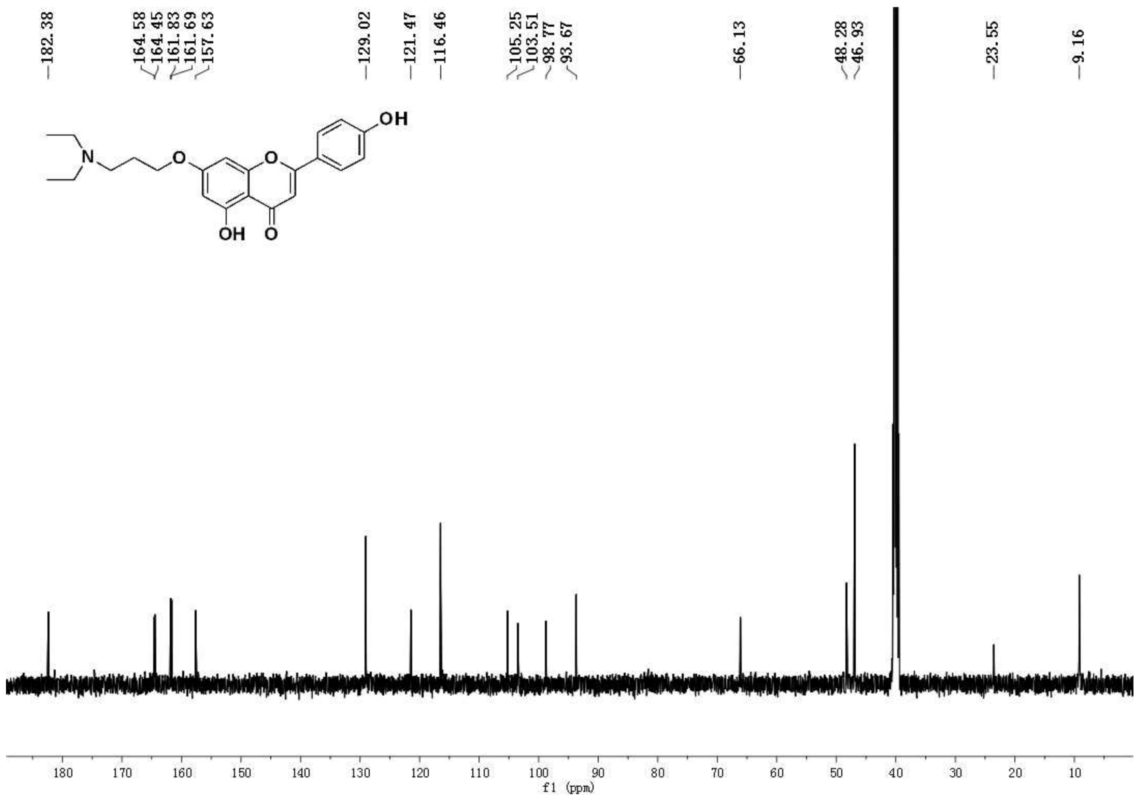

Figure S27. ^{1}H-NMR spectrum (400 MHz, DMSO-d_6) of 7-[3-(ethylamino)propoxy]-5-hydroxy-2-(4-hydroxyphenyl)-4H-chromen-4-one **4b.**

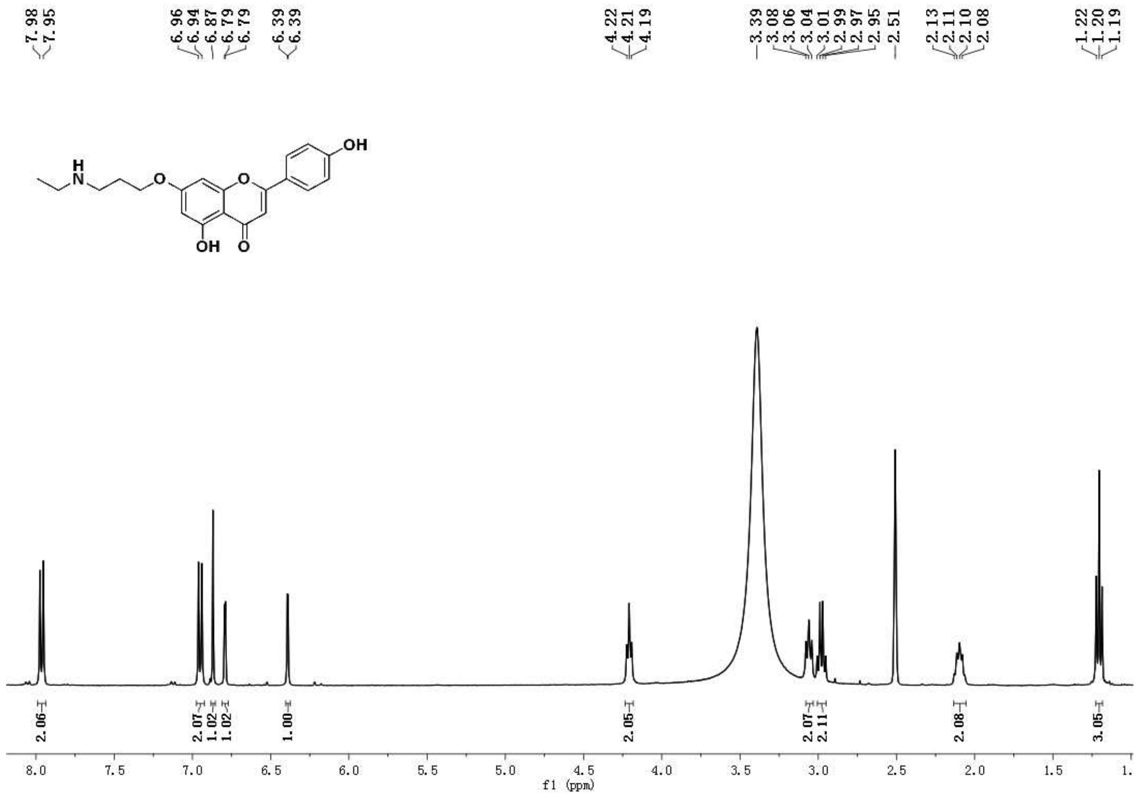

Figure S28. ^{13}C-NMR spectrum (100 MHz, DMSO-d_6) of 7-[3-(ethylamino)propoxy]-5-hydroxy-2-(4-hydroxyphenyl)-4H-chromen-4-one **4b.**

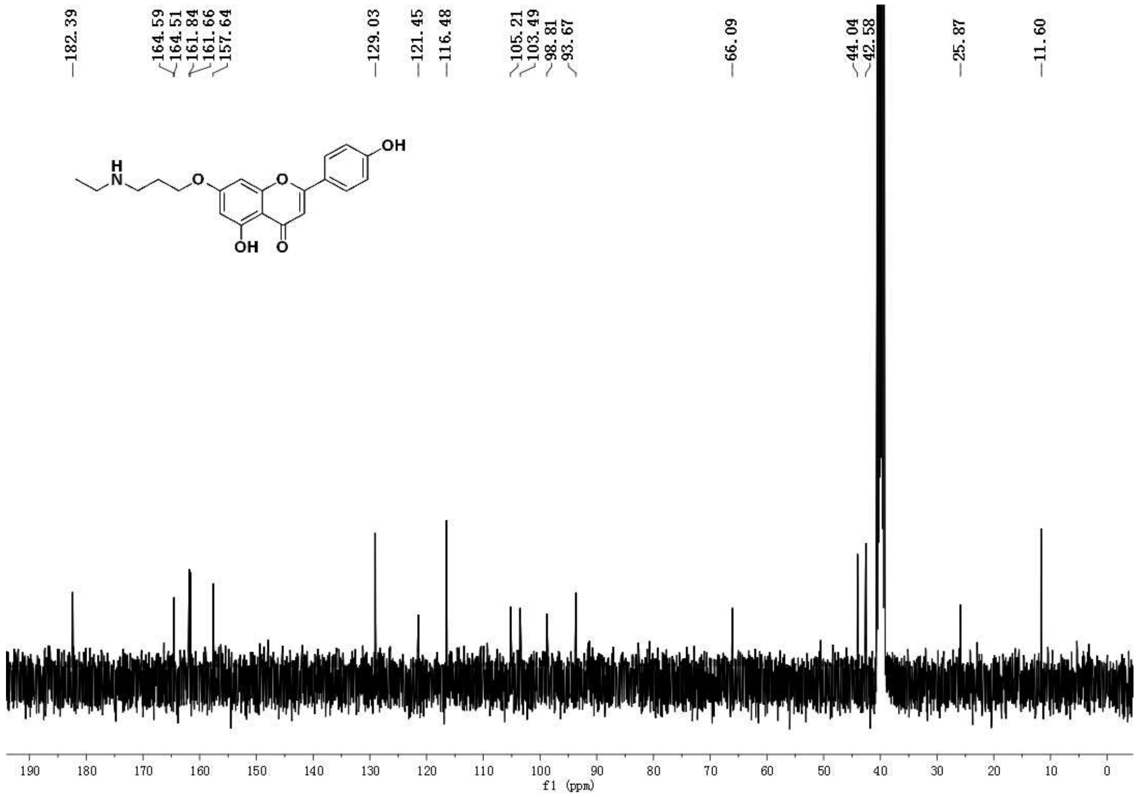

Figure S29. ^{1}H-NMR spectrum (400 MHz, DMSO-d_6) of 7-[3-(propylamino)propoxy]-5-hydroxy-2-(4-hydroxyphenyl)-4H-chromen-4-one **4c**.

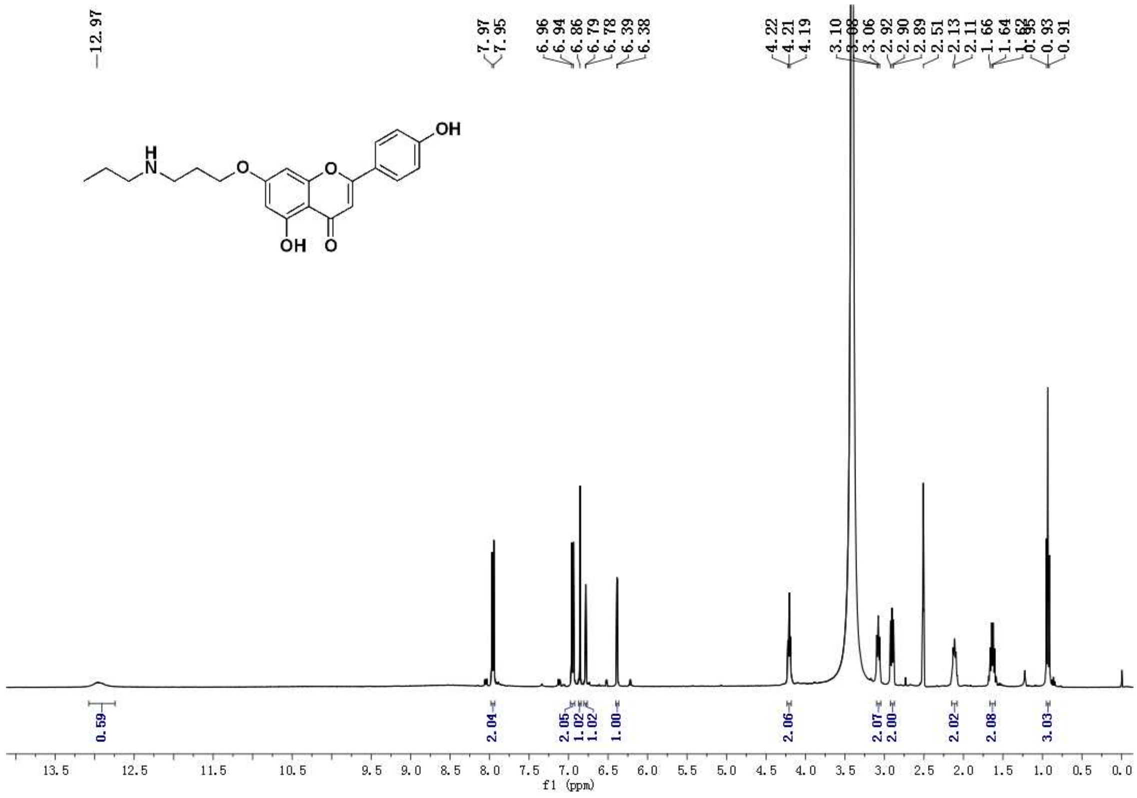

Figure S30. ^{13}C-NMR spectrum (100 MHz, DMSO-d_6) of 7-[3-(propylamino)propoxy]-5-hydroxy-2-(4-hydroxyphenyl)-4H-chromen-4-one **4c**.

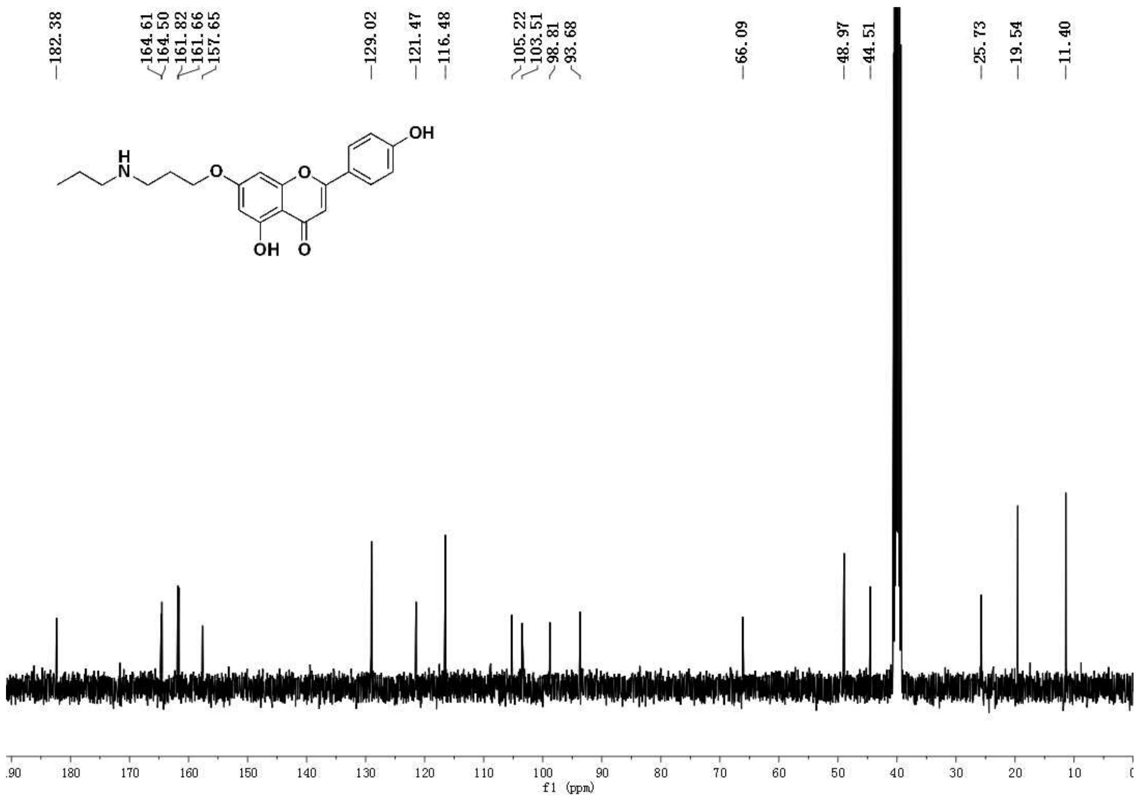

Figure S31. ^{1}H-NMR spectrum (400 MHz, DMSO-d_6) of 7-[3-(*n*-butylamino)propoxy]-5-hydroxy-2-(4-hydroxyphenyl)-4H-chromen-4-one **4d**.

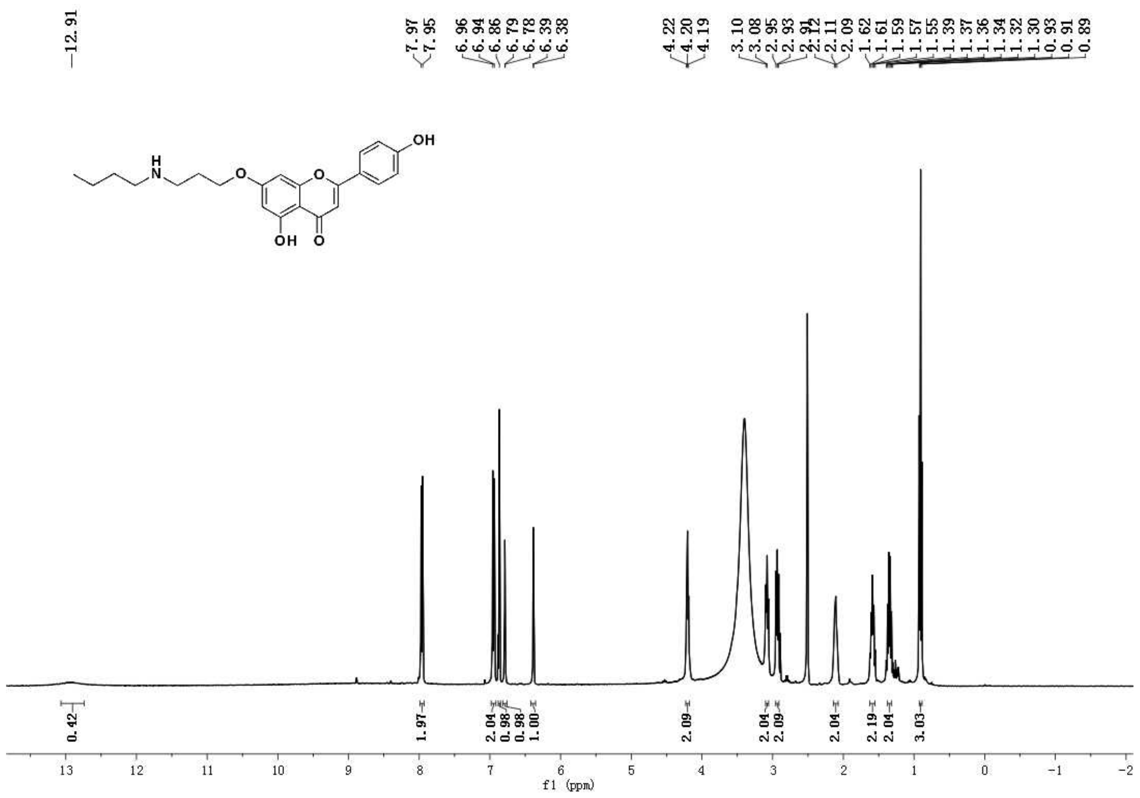

Figure S32. ^{13}C-NMR spectrum (100 MHz, DMSO-d_6) of 7-[3-(*n*-butylamino)propoxy]-5-hydroxy-2-(4-hydroxyphenyl)-4H-chromen-4-one **4d**.

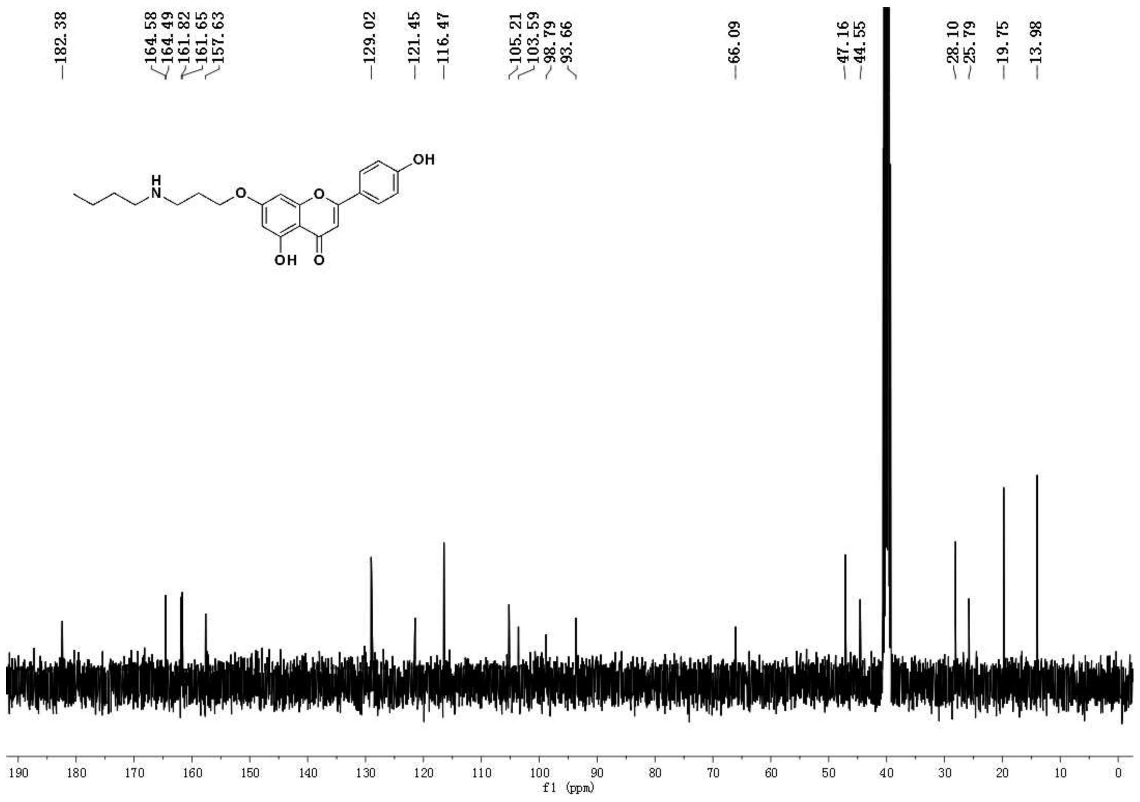

Figure S33. ^{1}H-NMR spectrum (400 MHz, DMSO-d_6) of 7-[3-(*tret*-butylamino) propoxy]-5-hydroxy-2-(4-hydroxyphenyl)-4H-chromen-4-one **4e**.

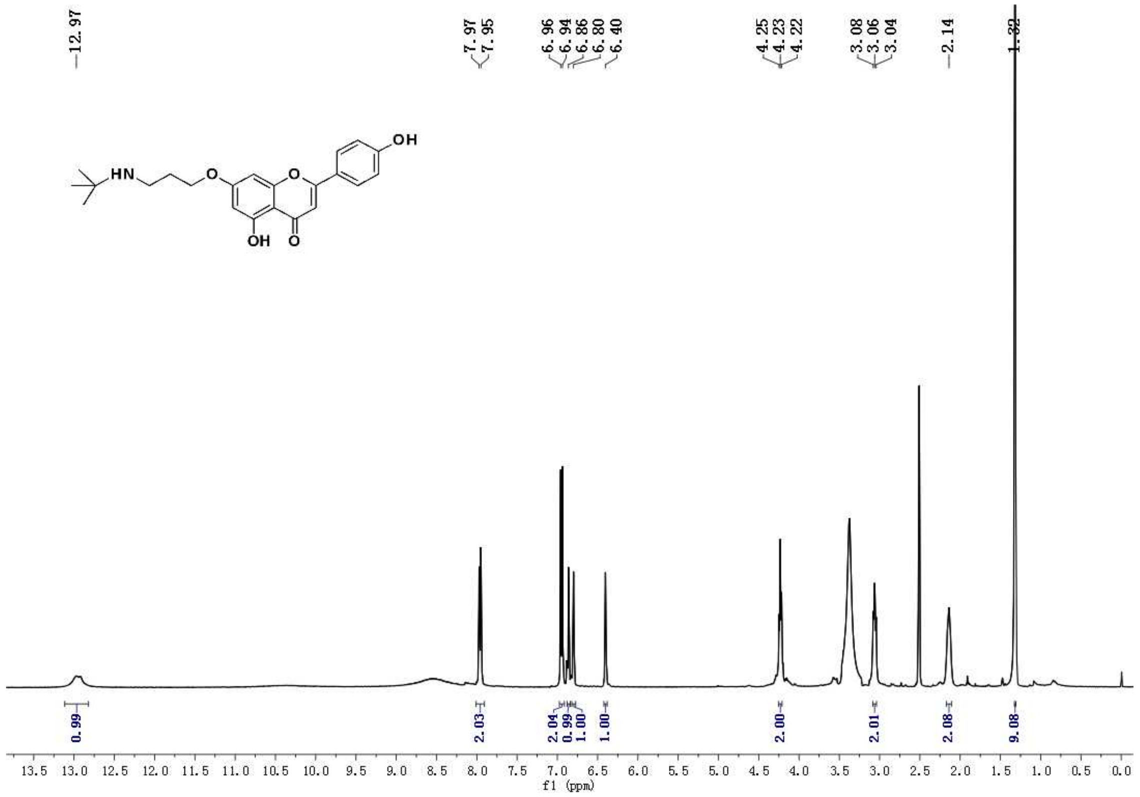

Figure S34. ^{13}C-NMR spectrum (100 MHz, DMSO-d_6) of 7-[3-(*tret*-butylamino) propoxy]-5-hydroxy-2-(4-hydroxyphenyl)-4H-chromen-4-one **4e**.

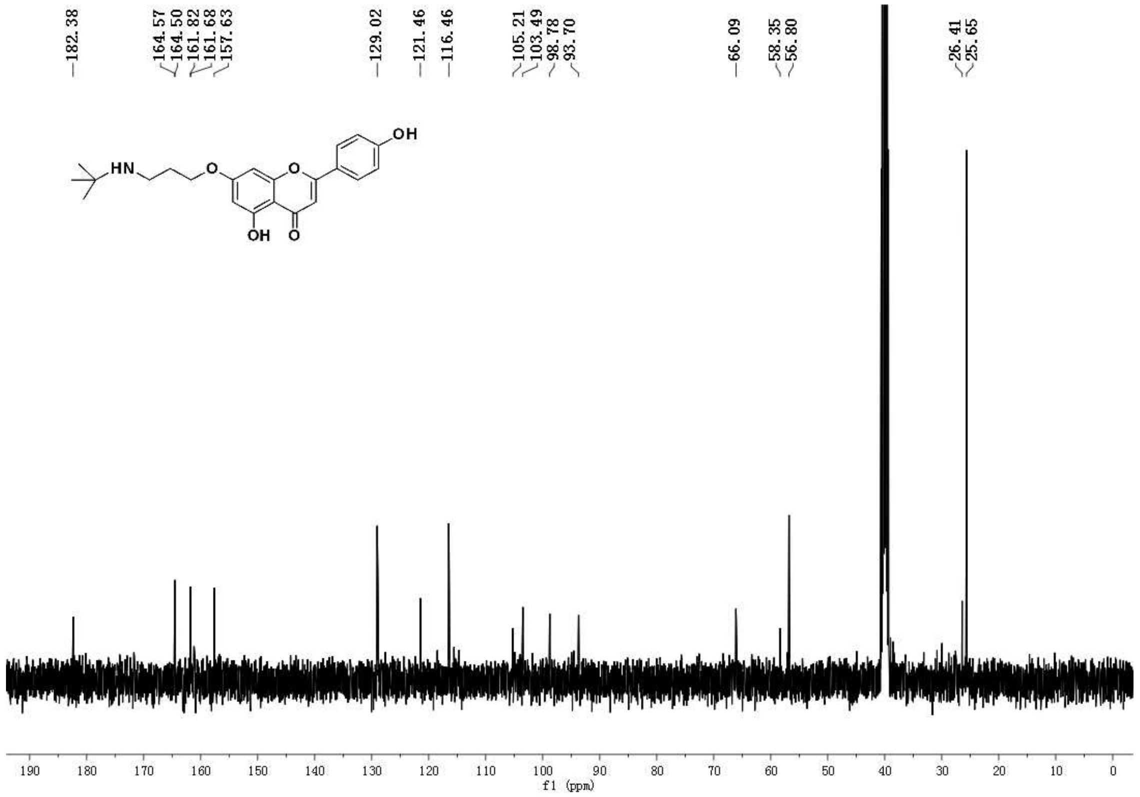

Figure S35. ^{1}H-NMR spectrum (400 MHz, DMSO-d_6) of 7-[3-(2′-hydroxyethylamino)propoxy]-5-hydroxy-2-(4-hydroxyphenyl)-4H-chromen-4-one **4f**.

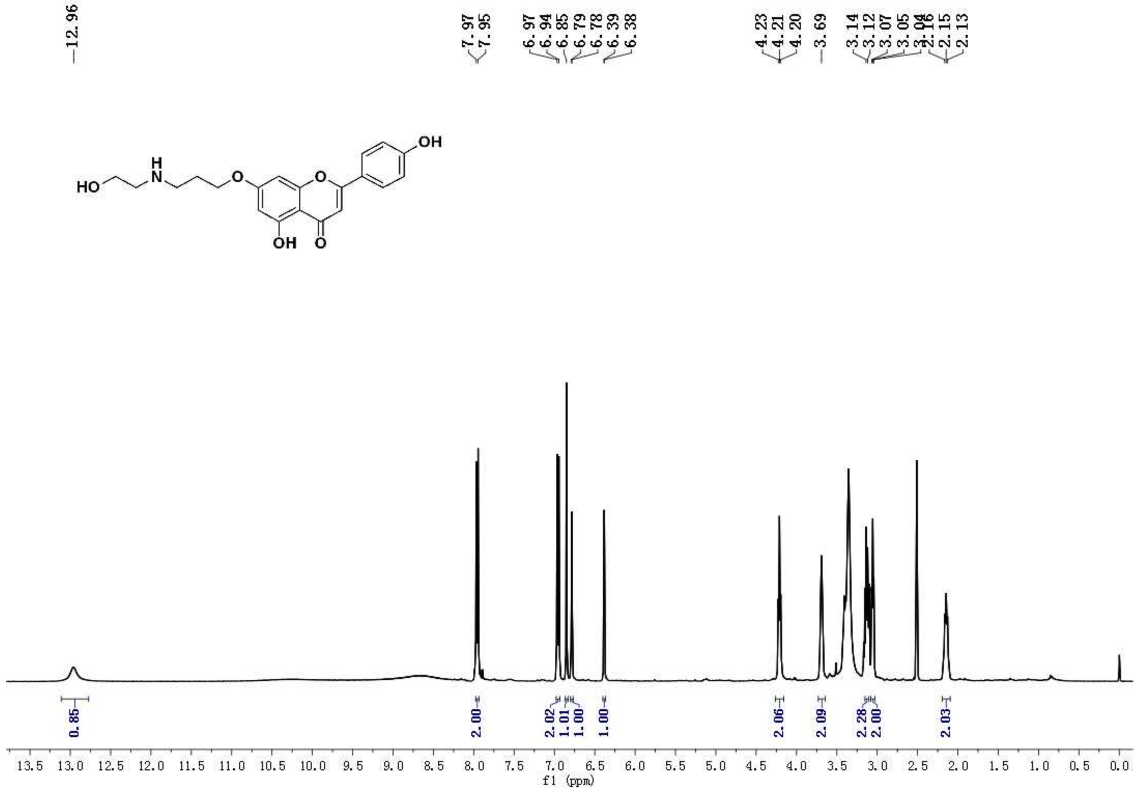

Figure S36. ^{13}C-NMR spectrum (100 MHz, DMSO-d_6) of 7-[3-(2′-hydroxyethylamino)propoxy]-5-hydroxy-2-(4-hydroxyphenyl)-4H-chromen-4-one **4f**.

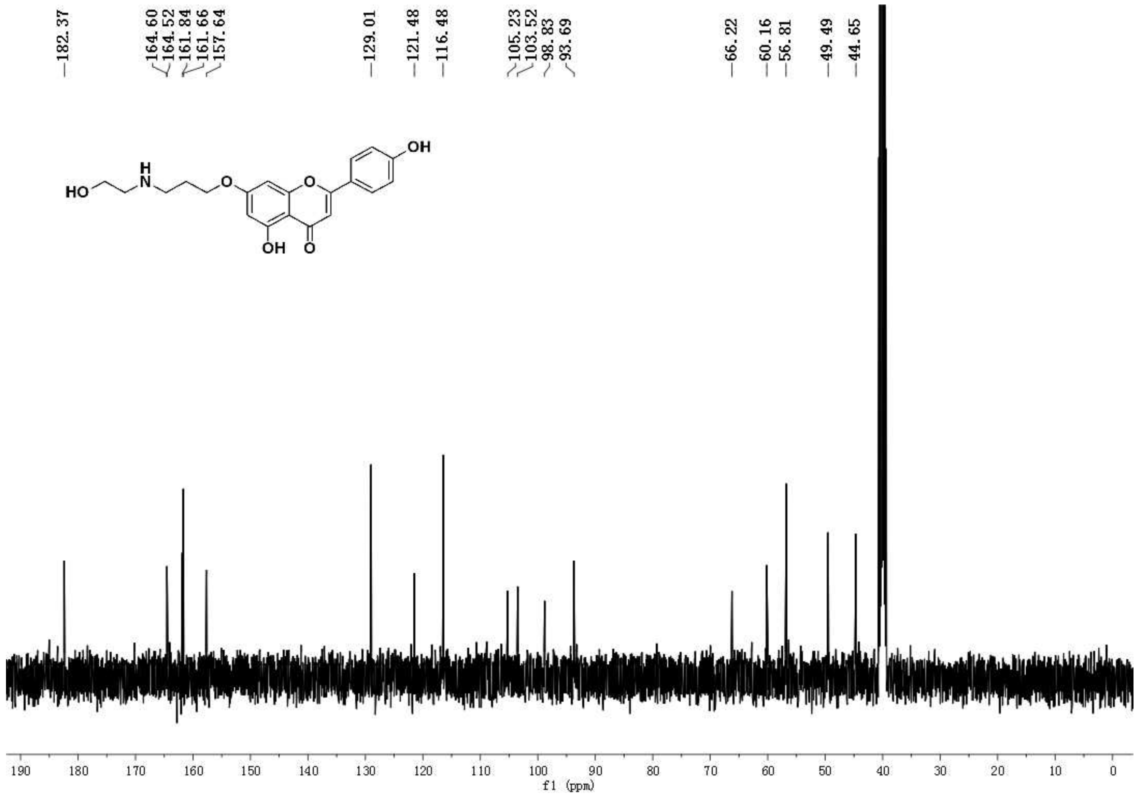

Figure S37. ^{1}H-NMR spectrum (400 MHz, DMSO-d_6) of 7-[3-(cyclohexylamino)propoxy]-5-hydroxy-2-(4-hydroxyphenyl)-4H-chromen-4-one **4g.**

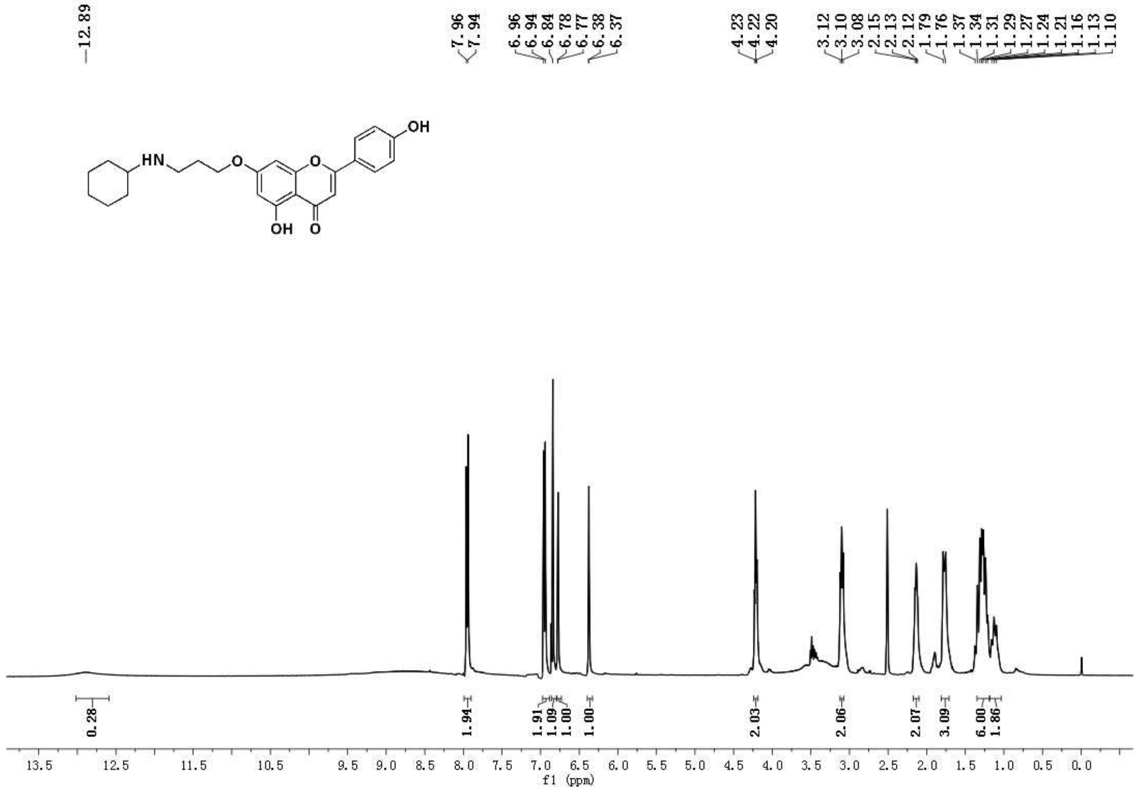

Figure S38. ^{13}C-NMR spectrum (100 MHz, DMSO-d_6) of 7-[3-(cyclohexylamino)propoxy]-5-hydroxy-2-(4-hydroxyphenyl)-4H-chromen-4-one **4g**.

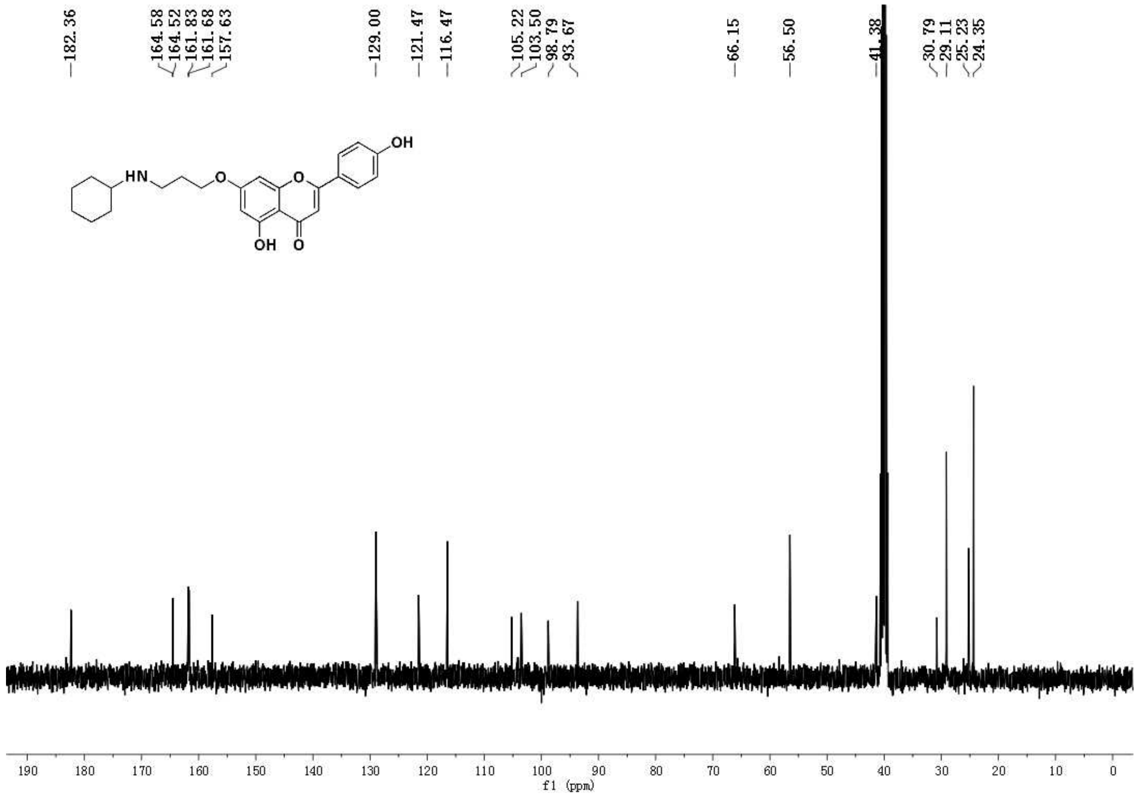

Figure S39. ^{1}H-NMR spectrum (400 MHz, DMSO-d_6) of 7-[3-(pyrrolidin-1-yl)propoxy]-5-hydroxy-2-(4-hydroxyphenyl)-4H-chromen-4-one **4h**.

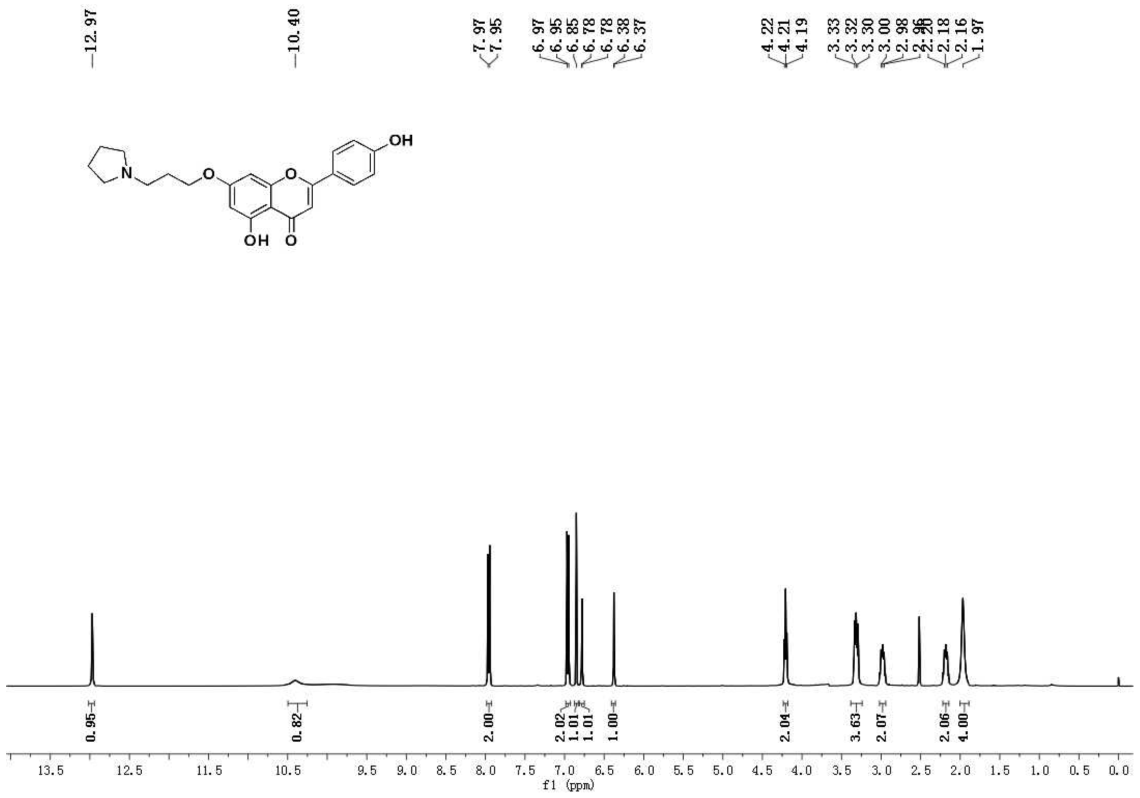

Figure S40. ^{13}C-NMR spectrum (100 MHz, DMSO-d_6) of 7-[3-(pyrrolidin-1-yl)propoxy]-5-hydroxy-2-(4-hydroxyphenyl)-4H-chromen-4-one **4h**.

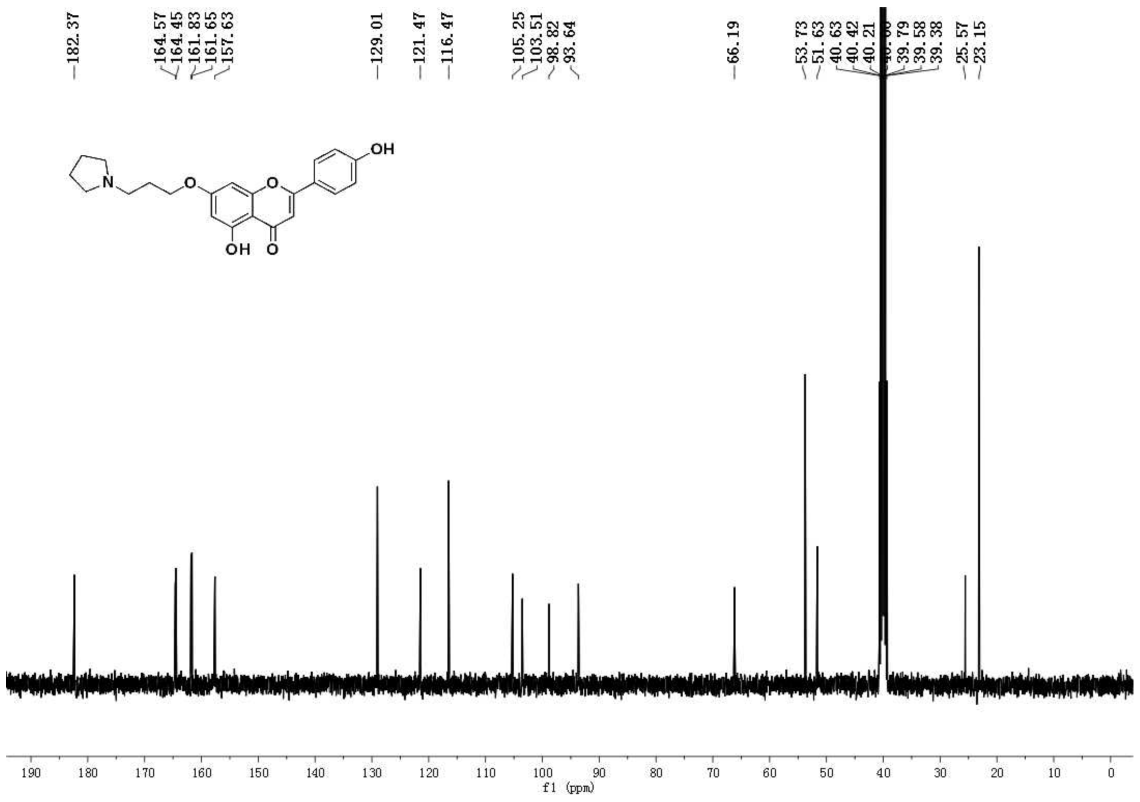

Figure S41. ^{1}H-NMR spectrum (400 MHz, DMSO-d_6) of 7-[3-(morpholin-4-yl)propoxy]-5-hydroxy-2-(4-hydroxyphenyl)-4H-chromen-4-one **4i**.

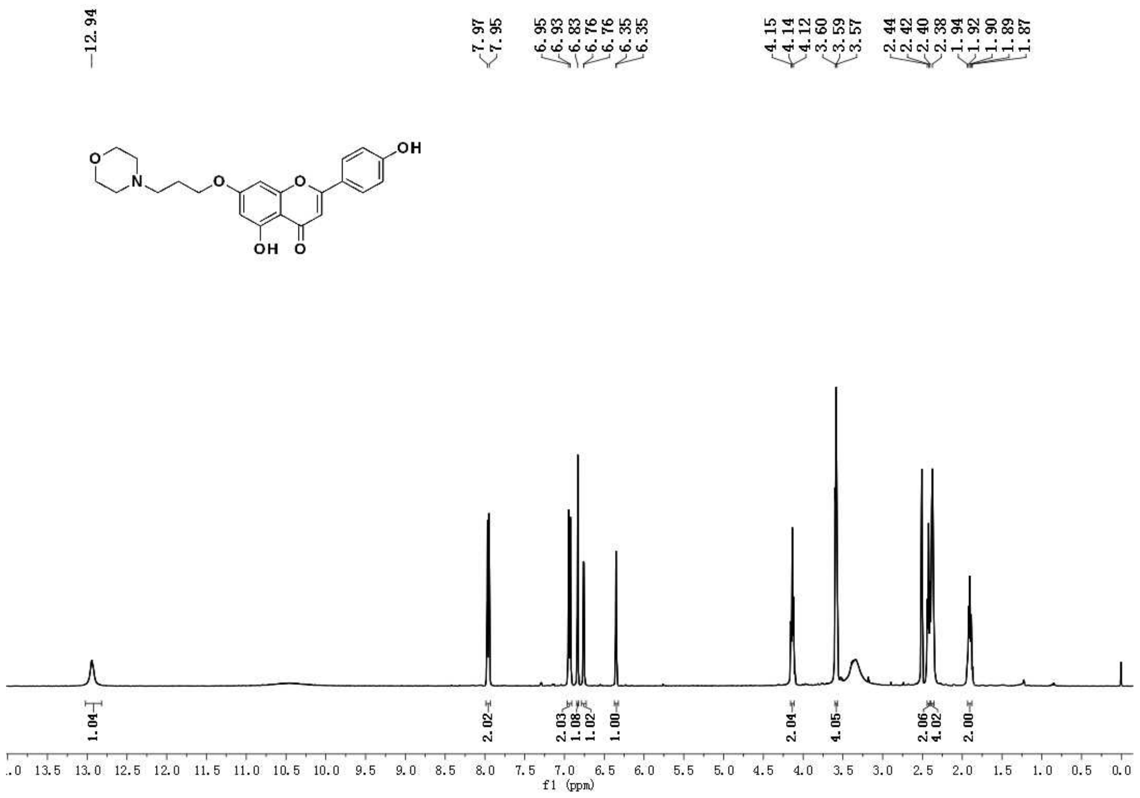

Figure S42. ^{13}C-NMR spectrum (100 MHz, DMSO-d_6) of 7-[3-(morpholin-4-yl)propoxy]-5-hydroxy-2-(4-hydroxyphenyl)-4H-chromen-4-one **4i**.

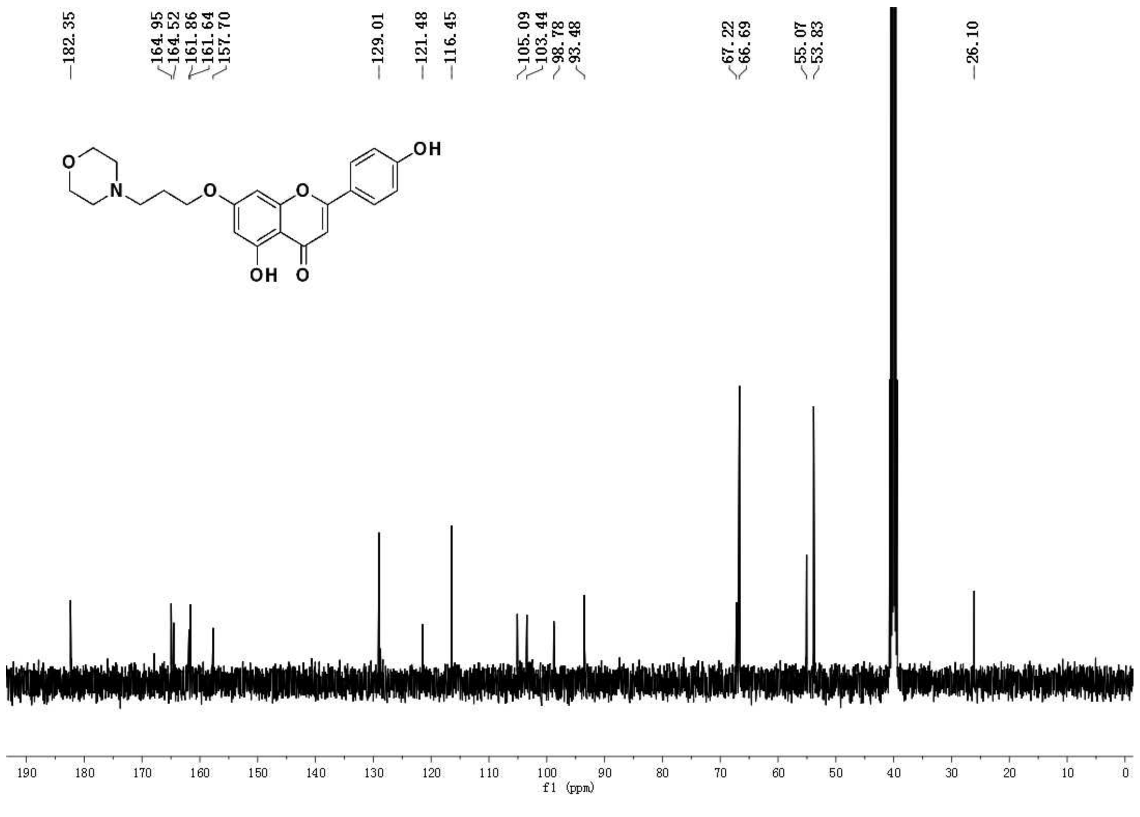

Figure S43. ^{1}H-NMR spectrum (400 MHz, DMSO-d_6) of 7-[3-(piperazin-1-yl)propoxy]-5-hydroxy-2-(4-hydroxyphenyl)-4H-chromen-4-one **4j**.

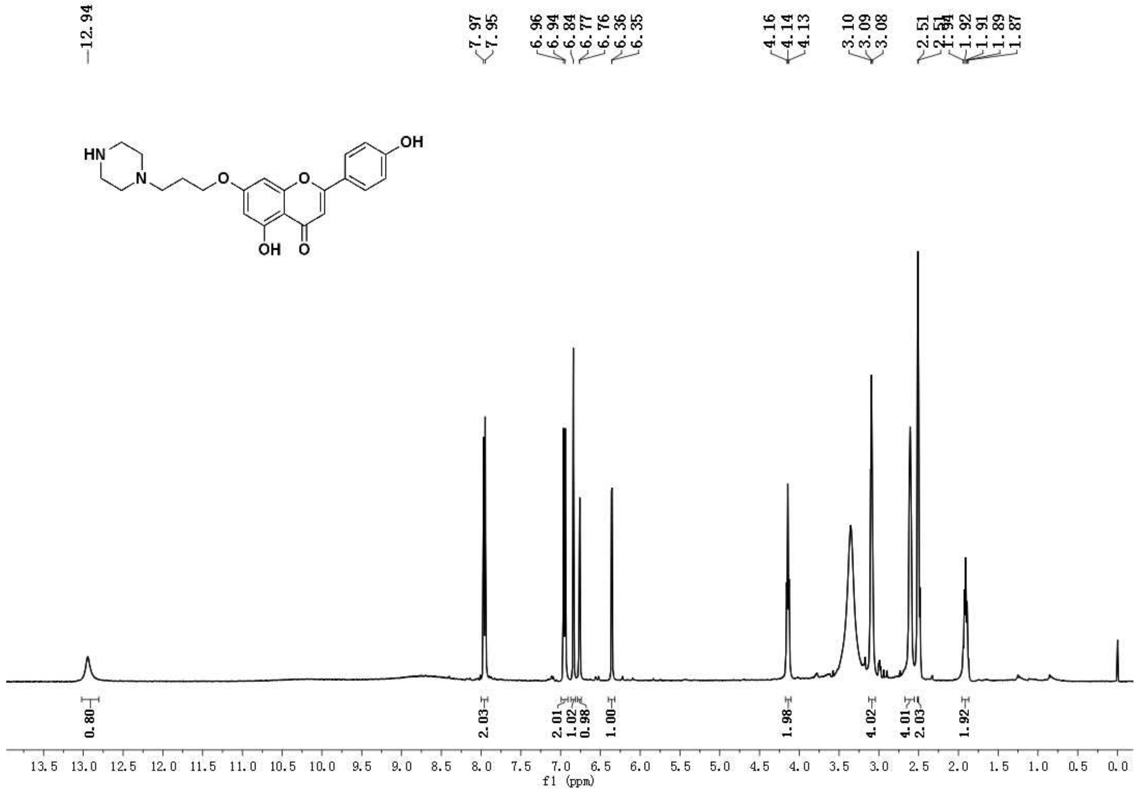

Figure S44. ^{13}C-NMR spectrum (100 MHz, DMSO-d_6) of 7-[3-(piperazin-1-yl)propoxy]-5-hydroxy-2-(4-hydroxyphenyl)-4H-chromen-4-one **4j**.

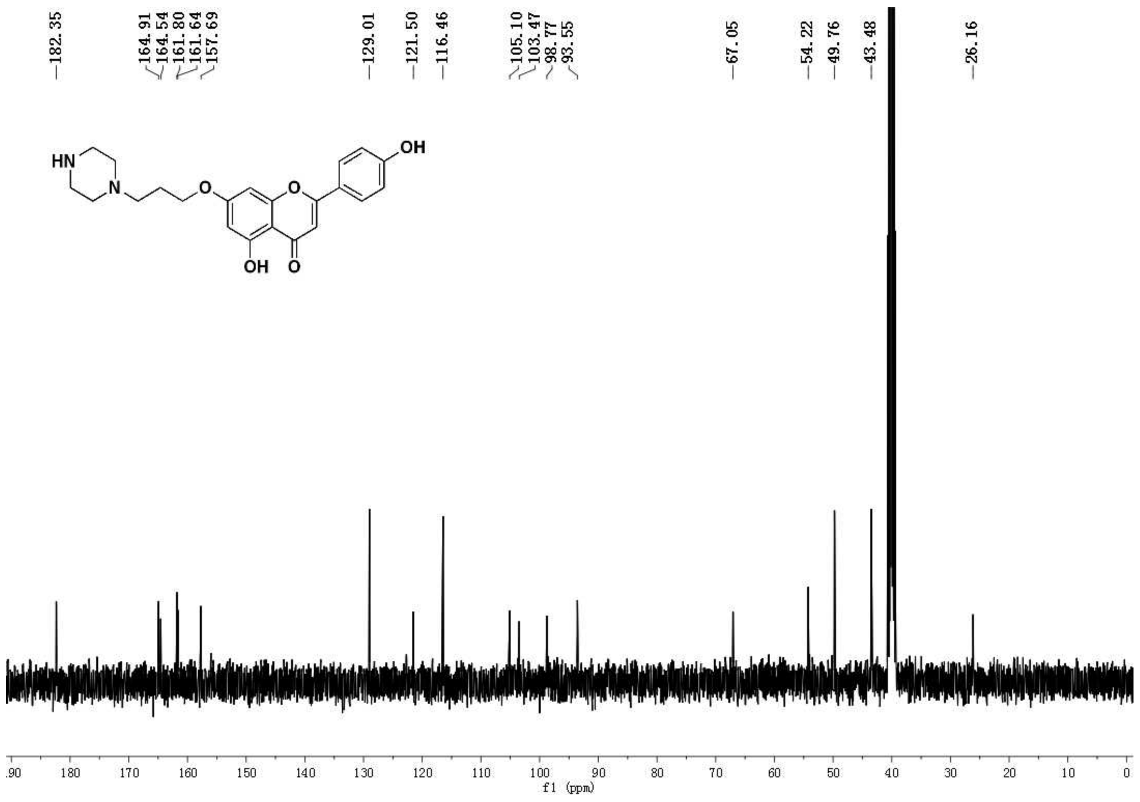

Figure S45. ^{1}H-NMR spectrum (500 MHz, DMSO-d_6) of apigenin (**1**).

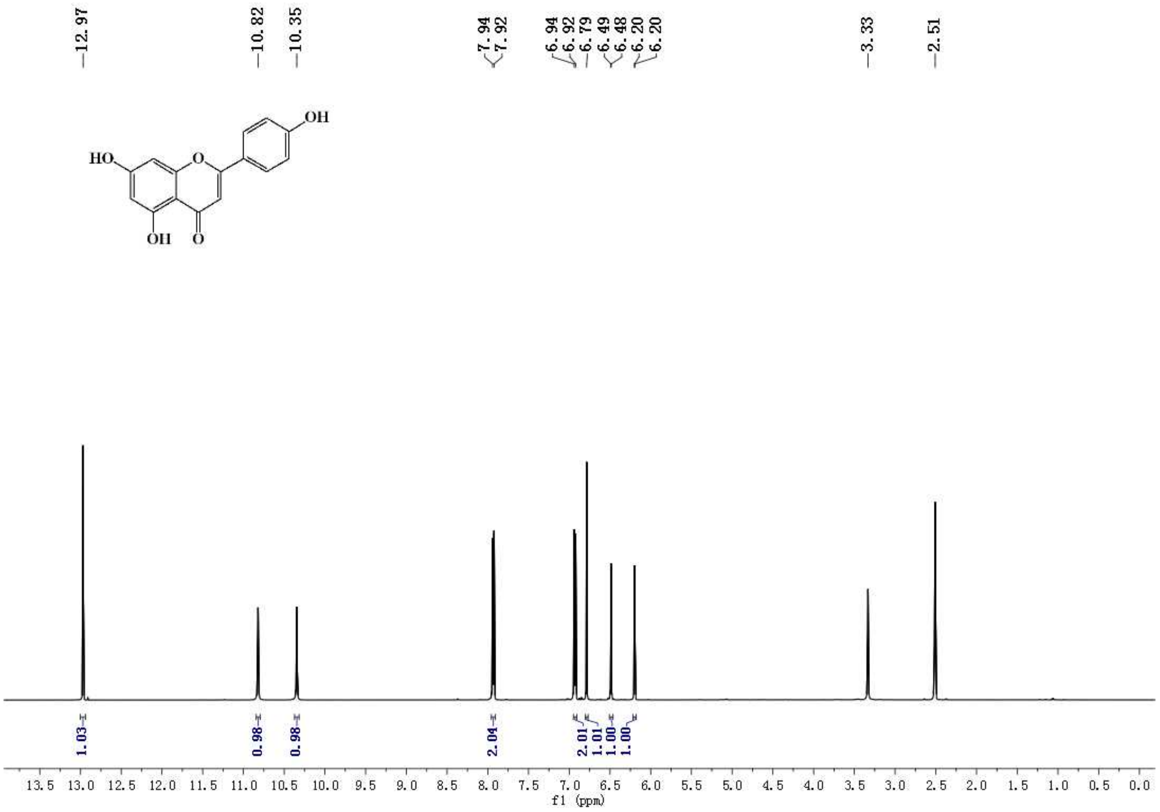

Figure S46. ^{13}C-NMR spectrum (125 MHz, DMSO-d_6) of apigenin (**1**).

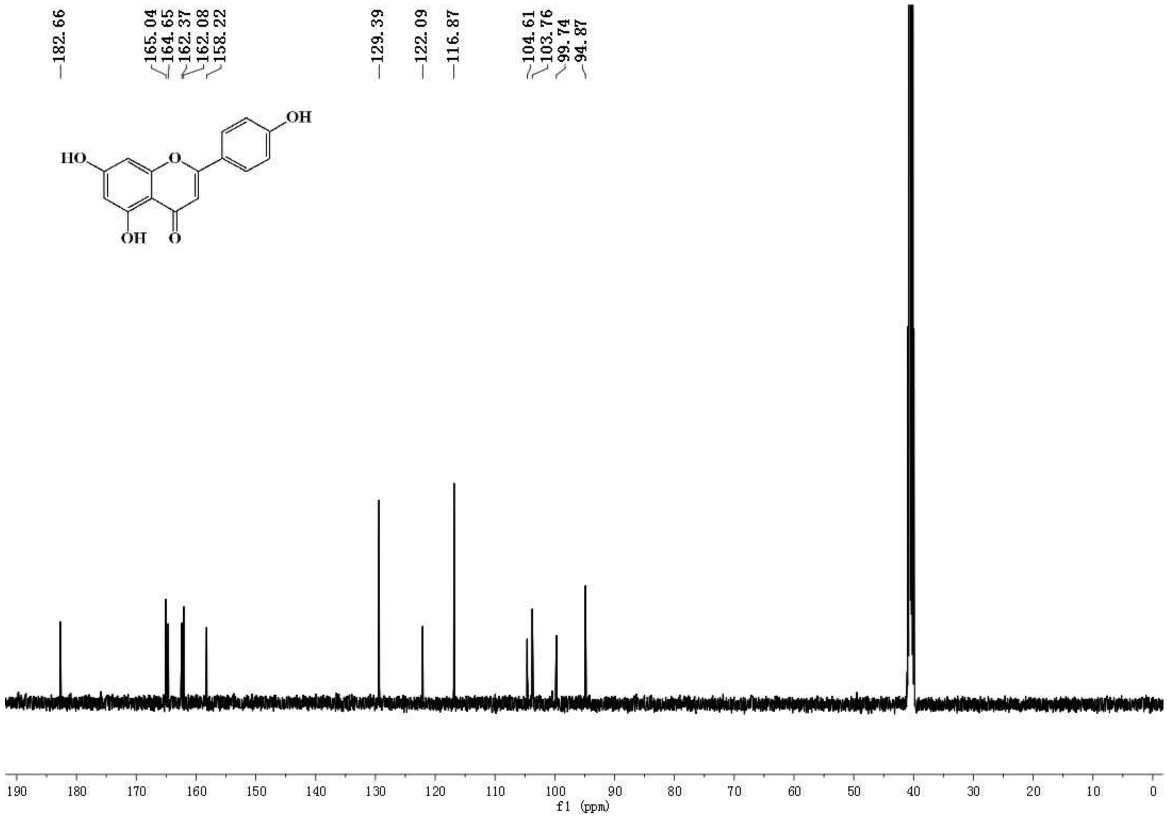

Supplement: Supplementary file 1 [file molecules-18-11496-s001.pdf]
